# Shortwave-infrared-light-emitting probes for the in vivo tracking of cancer vaccines and the elicited immune responses

**DOI:** 10.1038/s41551-023-01083-5

**Published:** 2023-08-24

**Authors:** Fuqiang Ren, Feifei Wang, Ani Baghdasaryan, Ying Li, Haoran Liu, RuSiou Hsu, Chuchu Wang, Jiachen Li, Yeteng Zhong, Felix Salazar, Chun Xu, Yingying Jiang, Zhuoran Ma, Guanzhou Zhu, Xiang Zhao, Kerry Kaili Wong, Richard Willis, K. Christopher Garcia, Anna Wu, Elizabeth Mellins, Hongjie Dai

**Affiliations:** 1 Department of Chemistry and Bio-X, Stanford University, Stanford, CA USA; 2 Department of Pediatrics, Human Gene Therapy, Stanford University, Stanford, CA USA; 3 Department of Molecular and Cellular Physiology, Stanford University, Stanford, CA USA; 4 Howard Hughes Medical Institute, Stanford University, Stanford, CA USA; 5 Department of Radiation Oncology, City of Hope, CA USA; 6 Departments of Molecular and Cellular Physiology and Structural Biology, Stanford University School of Medicine, Stanford, CA USA; 7 NIH Tetramer Facility at Emory Vaccine Center, Emory University, Atlanta, GA USA

**Keywords:** Cancer imaging, Protein delivery, Nanoparticles, Optical techniques

## Abstract

Tracking and imaging immune cells in vivo non-invasively would offer insights into the immune responses induced by vaccination. Here we report a cancer vaccine consisting of polymer-coated NaErF_4_/NaYF_4_ core–shell down-conversion nanoparticles emitting luminescence in the near-infrared spectral window IIb (1,500–1,700 nm in wavelength) and with surface-conjugated antigen (ovalbumin) and electrostatically complexed adjuvant (class-B cytosine–phosphate–guanine). Whole-body wide-field imaging of the subcutaneously injected vaccine in tumour-bearing mice revealed rapid migration of the nanoparticles to lymph nodes through lymphatic vessels, with two doses of the vaccine leading to the complete eradication of pre-existing tumours and to the prophylactic inhibition of tumour growth. The abundance of antigen-specific CD8^+^ T lymphocytes in the tumour microenvironment correlated with vaccine efficacy, as we show via continuous-wave imaging and lifetime imaging of two intravenously injected near-infrared-emitting probes (CD8^+^-T-cell-targeted NaYbF_4_/NaYF_4_ nanoparticles and H-2K^b^/ovalbumin_257-264_ tetramer/PbS/CdS quantum dots) excited at different wavelengths, and by volumetrically visualizing the three nanoparticles via light-sheet microscopy with structured illumination. Nanoparticle-based vaccines and imaging probes emitting infrared light may facilitate the design and optimization of immunotherapies.

## Abstract (web summary)

Multiplexed imaging of three types of infrared-light-emitting nanoparticle allows for the tracking of a nanoparticle-based cancer vaccine and of the elicited responses from intratumoural antigen-specific T lymphocytes in mice.

## Main

Activating the body’s own immune system to fight cancer through vaccination is at the frontier of cancer treatment and prevention, and can complement or replace traditional surgical resection, radiotherapy and chemotherapy. Notable progress has been made in the development of safe and efficient cancer vaccines^1^, including the US Food and Drug Administration-approved human papillomavirus vaccine to prevent cervical and other cancers, as well as several vaccines to treat existing cancers in patients^2–4^. Therapeutic cancer vaccines aim to treat existing cancer by activating the adaptive immune system and triggering T-lymphocyte responses, including the production of antigen-specific CD8^+^ cytotoxic T lymphocytes (CTLs) that can recognize and kill cancer cells. In contrast, prophylactic cancer vaccines are designed to induce antitumour immune responses and prevent the development of cancer. Lymph nodes, which contain large number of antigen-presenting cells (APCs) responsible for initiating and regulating adaptive immune responses, are a primary target for vaccine delivery and immunotherapy^5,6^. The ovalbumin (OVA) system has been widely employed to develop cancer vaccines in pre-clinical models, using a wide range of delivery vehicles, including inorganic nanoparticles^7^ and lipid nanoparticles^8^, to prophylactically prevent or therapeutically treat OVA-expressing E.G7 mouse lymphoma tumours. To improve vaccine efficacy, researchers have co-administrated OVA antigen with adjuvants such as CpG oligodeoxynucleotides^9–11^. CpG B, a toll-like receptor ligand that binds to TLR9 expressed on the endosomal membrane, directly activates dendritic cells (DCs), macrophages and B cells, driving strong T helper 1 effector responses and enhancing T-cell cytotoxicity^3,12^. Several nanoparticles have been used to co-deliver CpG B and OVA for tumour treatment^9,11,13–15^, but with low long-term survival rates and small treatable tumour sizes (<~200 mm^3^ in volume)^14,15^. In addition, previous vaccine development typically lacked in vivo imaging of vaccine trafficking and lymph node targeting^16,17^. In vivo longitudinal imaging/monitoring of immune responses to vaccination has also been rare for correlating with therapeutic outcomes.

Spatially and temporally resolved imaging of an administrated vaccine, and in vivo assessment of the immune cell responses, could lead to new understandings of the immune system and guide vaccine design. With reduced light scattering and near-zero autofluorescence, in vivo near-infrared-II/short-wave infrared (NIR-II/SWIR, 1,000–3,000 nm) fluorescence imaging through intact mouse skin and tissues allows for the visualization of biological structures and their dynamics down to single-cell resolution and at depths of millimetres^18–29^. For example, in vivo NIR-II structured-illumination light-sheet microscopy (LSM-SIM) can longitudinally map out cellular CD4, CD8 and OX40 expression levels in tumour tissue in response to immunotherapy^27^. Recently, the excitation and emission wavelengths of one-photon confocal fluorescence microscopy were extended to ~1,650 nm and beyond 1,700 nm (NIR-IIc, 1,700–2,000 nm) respectively, enabling non-invasive in vivo molecular imaging of high endothelial venules, CD169^+^ subcapsular sinus macrophages and CD3^+^ T cells in mouse lymph nodes with single-vessel and single-cell resolution^25^. For vaccine/immunotherapy studies of mouse models, in vivo NIR-II imaging using wide-field and LSM/confocal microscopy modes offers new opportunities for dynamically monitoring vaccine trafficking as well as immune cell responses in tumours and draining lymph nodes over time. Such capabilities could add a new dimension to conventional ex vivo flow cytometry analysis of immune cells (such as CD8^+^ CTLs) extracted from various organs/tissues.

In this Article, we report the development of a nanovaccine using a synthesized NIR-IIb luminescent nanoparticle, pErNP, as both an NIR-II/SWIR imaging tracer and vaccine carrier for the OVA antigen and the CpG B adjuvant. Therapeutically, two subcutaneous (s.c.) administrations of the pErNP–OVA–CpG B vaccine resulted in 100% eradication of E.G7 tumours and increased mouse survival (*n* = 17). Additionally, this vaccine prophylactically protected immunized healthy mice against tumour growth. The NIR-IIb luminescence of pErNP–OVA–CpG B allowed dynamic imaging of the vaccine’s migration through lymphatic vessels and lymph-node targeting and homing. We also employed two additional NIR-IIb probes, quantum dots (QDs) and doped Er nanoparticles, which we named ‘ErNP’, for in vivo three-plex molecular imaging of antigen-specific CD8^+^ CTLs in both wide-field and high-resolution microscopy modes to resolve CTLs in the tumour microenvironment. Such imaging was performed longitudinally within minutes of administration and up to several weeks. The results show that NIR-II/SWIR nanoprobes offer unique opportunities for guiding vaccine design and for investigating immune responses with unprecedented spatial and temporal resolution over a long time span.

## Results

### Synthesis of pErNP for vaccine carrier and NIR-II/SWIR imaging in the 1,500–1,700 nm range

Previously, we developed a cubic α-phase down-conversion nanoparticle composed of 2% Er, 2% Ce, 10% Zn doped NaYbF_4_ core/NaYF_4_ shell^30^ (ErNP), and PbS/CdS core/shell QDs^20^ emitting in the 1,500–1,700 nm range for two-plex molecular imaging with millimetres tissue penetration depth, high signal/background ratio and near-zero autofluorescence^18,25,30^. The much longer luminescence lifetime of ErNP (>4 ms) than that of QD (~46 µs) was utilized for two-plex imaging in continuous-wave (CW) and lifetime modes, allowing differentiation of the two probes emitting in the same NIR-IIb range^30^.

Here we aimed to increase imaging multiplexity by synthesizing a pErNP composed of a pure hexagonal β-phase NaErF_4_ core (without other rare-earth dopants, hence the name ‘pure ErNP’ or pErNP) with a NaYF_4_ shell (Supplementary Fig. 1; synthesis details in Methods). Transmission electron microscopy (TEM) imaging revealed a uniform size of the pErNP with a mean diameter of ~21.7 nm (Supplementary Fig. 2a,b) and clear lattice fringes corresponding to the 0.513 nm *d*-spacing of (100) planes (Supplementary Fig. 2c,d). We clearly resolved a ~3.8 nm shell on the pErNP (Supplementary Fig. 2c,d), which was known to protect the nanocrystal core from luminescence quenching by water molecules in aqueous solutions^30,31^.

**Fig. 1:**
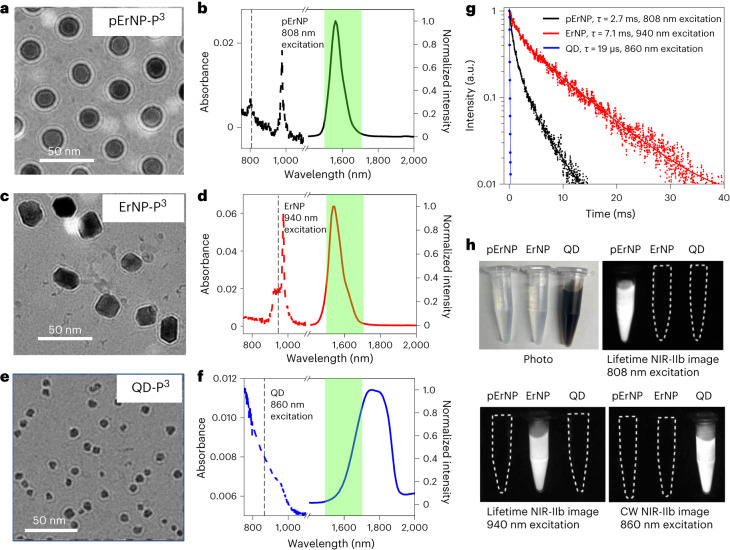
Three nanoparticle probes luminescence/emitting in the 1,500–1,700 nm range for vaccine carrier and multiplexed NIR/SWIR imaging. **a**,**c**,**e**, Cryo-EM images of pErNP-P^3^ (**a**), ErNP-P^3^ (**b**) and QD-P^3^ (**e**) in PBS buffer. These images show a uniform coating of approximately 5 nm thickness on the three types of particle. **b**,**d**,**f**, Absorption (dashed curves) and emission (solid curves) spectra of three different nanoparticles, pErNP-P^3^ (**b**), ErNP-P^3^ (**d**) and QD-P^3^ (**f**). The green shaded areas are the NIR-IIb 1,500–1,700 nm detection range for imaging. The dashed vertical lines were drawn to show the excitation wavelengths (808 nm for pErNP, 940 nm for ErNP and 860 nm for QD) used for three-plex imaging for each of the three probes in lifetime (for pErNP and ErNP) or CW (for QD) mode. **g**, Lifetime decays of pErNP, ErNP and QD, respectively. In **b**, **d** and **f**, pErNP and ErNP were measured in cyclohexane, QD was measured in PBS buffer, and photoluminescence (PL) decay curves were fit with a typical bi-exponential function. **h**, A colour photograph and multiplexed NIR-IIb imaging of pErNP, ErNP and QD in PBS buffer under three different imaging conditions that detect one probe at a time without imaging the other two. pErNP: 808 nm excitation, 1,500–1,700 nm detection in lifetime mode (for three-plex imaging details, see Supplementary Information). ErNP: 940 nm excitation, 1,500–1,700 nm detection in lifetime mode; QD: 860 nm excitation, 1,500–1,700 nm in CW.

Under excitation at 808 nm or 975 nm, the pErNP (with a pure NaErF_4_ nanocrystal core) exhibited substantial light adsorption by the Er ions (Fig. 1b). The resulting down-conversion luminescence emission^30,31^ was in the 1,500–1,700 nm NIR-IIb range (Fig. 1b) and had a long-lived luminescence lifetime of ~2.7 ms (Fig. 1g). In comparison, our previously reported ErNPs, which emitted in the NIR-IIb range (~20.6 nm size, Supplementary Fig. 3a–c), had a different absorption peak at ~900–980 nm and a ~7.1 ms emission lifetime (Fig. 1d,g). The PbS/CdS QD had a size of ~7.7 nm (Supplementary Fig. 3d–f) and broad absorption (Fig. 1f,g). Although all three probes emitted in the same NIR-IIb range of 1,500–1,700 nm, we were able to differentiate them for multiplexed imaging by using lifetime imaging of pErNPs after a pulsed 808 nm laser excitation, lifetime imaging of ErNPs after a pulsed 940 nm laser excitation, and 860 nm CW QD imaging, respectively (Fig. 1h, Table 1 and Methods). This approach enabled multiplexed imaging of one of the three NIR-IIb probes without any crosstalk signals from the other two (Fig. 1h and Table 1). Note that the ErNPs absorbed light at ~940 nm (Fig. 1d) mainly by Yb ions in the 2% Er, 2% Ce and 10% Zn doped NaYbF_4_ core, with the excited Yb ions transferring energy to Er ions for NIR-IIb emission through down-conversion^30^. Pure Er ions in the core of pErNP absorbed little light at 940 nm (but absorbed at ~800 nm and ~975 nm, Fig. 1b), and gave no emission when imaging ErNPs under 940 nm excitation (Fig. 1h and Table 1). Multiplexed imaging with three different probes emitting in the same NIR-IIb 1,500–1,700 nm range without any crosstalk signals is substantial since it enables superior penetration depth and signal-to-background ratios for the imaging targets of all three probes in vivo. The minimized light scattering and autofluorescence in the NIR-IIb window can be utilized for multiple molecular targets in a single imaging experiment.

**Table 1:** Excitation conditions for pErNPs, ErNPs and PbS/CdS QDs, respectively

1,500–1,700 nm emission	Lifetime imaging excitation 808 nm	CW imaging excitation 860 nm	Lifetime imaging excitation 940 nm
pErNP	Yes	No	No
ErNP	No	No	Yes
QD IIb	No	Yes	No

For three-plex NIR-IIb imaging, without crosstalk of emission in the same 1,500–1,700 nm range.

To make the probes biocompatible, all three NIR-IIb emitting probes were transferred into aqueous solutions by cross-linking three layers of hydrophilic polymers (named ‘P^3^ coating’: branched polyethylene glycol/polyacrylic acid/polyethylene glycol) on the surfaces of the particles; see Methods)^24,30^. Cryogenic electron microscopy (cryo-EM) revealed a uniform ~5-nm-thick P^3^ layer on the particles (Fig. 1a,c,e). This surface coating allowed near-complete excretion of the resulting P^3^-nanoparticles from the body within 2 weeks post-administration without causing any adverse health effects^24,30^. The hydrodynamic sizes measured by dynamic light scattering (DLS) were ~52 nm, ~37 nm and ~35 nm for pErNP-P^3^, ErNP-P^3^ and QD-P^3^, respectively (Supplementary Fig. 4). The amino functional groups on the outmost P^3^ coating layer were utilized to either conjugate to antigens for vaccine formulation (using pEr-P^3^) or antibodies or MHC-I tetramers (using ErNP-P^3^ and QD-P^3^) for in vivo molecular imaging in NIR-IIb.

### pErNP–OVA–CpG B complex as a trackable vaccine by NIR-II/SWIR imaging

**Fig. 2:**
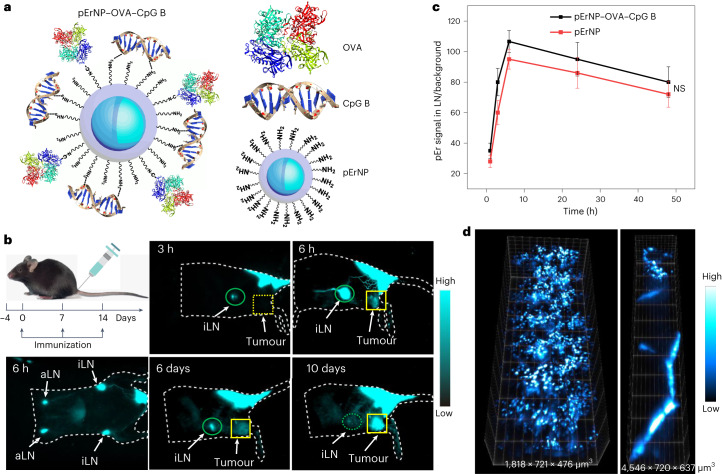
An NIR-II/SWIR emitting pEr nanoparticle for in vivo trackable nanovaccine. **a**, Schematic of a nanovaccine pErNP–OVA–CpG B (OVA proteins were conjugated to the NH_2_- groups on pErNP-P^3^ via EDC chemistry and then mixed with CpG B for electrostatic complexation to form the pErNP–OVA–CpG B nanocomplex). **b**, Immunization schedule, C57BL/6 mice were immunized by s.c. injection at the mouse tail base with the pErNP–OVA–CpG B nanovaccine on days 0, 7 and 14. Also shown are NIR-IIb luminescence images (the imaging conditions were as follows: excitation at 975 nm with a power density of approximately 50 mW cm^−2^ and detection at 1,500–1,700 nm, and all images were captured using an exposure time of 20 ms, except for the 6 h timepoint, where an exposure time of 50 ms was used to visualize the lymphatic vessels; the imaging was conducted in CW mode) showing the vaccine trafficking pathways after s.c. injection at the tail base of a mouse. Images were recorded at different timepoints as indicated p.i. **c**, The pErNP signal in iLN normalized by background plotted as a function of time post s.c. injection of pErNP–OVA–CpG B nanovaccine or pEr nanoparticle only (*P* = 0.5247). Data are presented as mean ± standard deviation and analysed by two-tailed Student’s *t*-test. **d**, Three-dimensional volumetric NIR-II SIM images of pErNP–OVA–CpG B in iLN (left) and lymphatic vessels (right). Imaging conditions: 975 nm excitation, 1,500–1,700 nm detection, exposure times 100 ms, CW mode.

We chose the pErNP-P^3^ as a vaccine carrier due to its hydrodynamic size of ~50 nm measured by DLS, in a range suitable for lymph node homing/trafficking^32,33^ to trigger adaptive immune responses. We conjugated whole OVA proteins (~45 kD) from chicken egg to the NH_2_-groups on pErNP-P^3^ using 1-(3-dimethylaminopropyl)-3-ethylcarbodiimide hydrochloride (EDC) chemistry and then simply mixed pErNP–OVA with CpG B to form the pErNP–OVA–CpG B nanocomplex through electrostatic interactions (Fig. 2a; see Methods). DLS and zeta-potential measurements (Supplementary Fig. 5) showed that the P^3^-coated pErNPs possessed a positive surface charge due to surface NH_2_-groups with a positive zeta-potential of ~7.3 mV in a pH 7.4 phosphate-buffered saline (PBS) buffer. After OVA conjugation, the zeta-potential decreased to ~6.1 mV, and after complexation with CpG B, the zeta-potential further decreased to ~3.8 mV. Upon conjugation of OVA and CpG B, the particle size slightly increased (Supplementary Fig. 5) from the initial ~53 nm (pErNP-P^3^) to ~57 nm (pErNP–OVA) and to ~59 nm (pErNP–OVA–CpG B).

The OVA to pErNP conjugation efficiency was quantified to be ~95.6%, without obvious detachment after a 24 h storage period in PBS buffer (detailed information in Supplementary Information and Supplementary Fig. 6). The complexation of CpG B with pErNP–OVA was confirmed using a fluorescently labelled CpG B, with ~68% of the fluorescein isothiocyanate (FITC)–CpG B remaining bound to pErNP–OVA even after rigorous washing (Supplementary Fig. 7).

### In vivo NIR-IIb imaging/tracking of administrated pErNP–OVA–CpG B nanovaccine

The pErNP–OVA–CpG B nanocomplexes were subcutaneously injected at the tail base of C57BL/6 mice bearing OVA expressing E.G7 tumours. The pErNPs were exploited not only as an antigen/TLR9 adjuvant delivery vehicle but also as an NIR-II/SWIR imaging agent for visualizing the vaccine trafficking pathway. In vivo wide-field NIR-IIb imaging (excitation ~975 nm or 808 nm; see Fig. 1b, emission 1,500–1,700 nm) showed a gradual increase of the pEr emission in the inguinal lymph node (iLN) over time, reaching a substantial level within ~3 h (Fig. 2b) post injection (p.i.) and peaking at ~6 h p.i. (Fig. 2b,c). Some of the nanovaccines remained at the s.c. injection site forming a depot (Fig. 2b), gradually decreasing/releasing over days and up to ~2 weeks. Supplementary Video 1 clearly showed the pErNP–OVA–CpG B vaccine trafficking through lymphatic vessels between the injection site, iLN and axillary lymph node (aLN), an important process for inducing immune responses. For higher resolution, we also performed three-dimensional (3D) volumetric LSM-SIM imaging to visualize the distribution of the pErNPs within the iLN and lymphatic vessels (Fig. 2d). Moreover, our NIR-LSM imaging captured the movement of the vaccine in the lymphatic vessels trafficking to the iLN (Supplementary Video 2).

One day after vaccinating mice (*n* = 3) with pErNP–OVA–CpG B, we collected the iLNs and used fluorescence-activated cell sorting (FACS) to isolate DCs (CD11c^+^), B cells (CD19^+^), T cells (CD3^+^) and macrophages (F4/80^+^) (Supplementary Fig. 8 and Supplementary Table 1). We imaged the pEr luminescent emission from the cells in the NIR-IIb window and observed pEr signals in all four types of cell in the iLN (Supplementary Fig. 9). For accurate quantification, the pEr content in each cell type was measured using Inductively Coupled Plasma Optical Emission spectroscopy (ICP-OES) (Supplementary Fig. 10 and Supplementary Table 2). We found that the DCs, macrophages and B cells had a per-cell-based pEr content of 13.6×, 101× and 2.1× fold higher than that of T cells, respectively. This suggested that APCs in the lymph nodes were effective in taking up the pErNP–OVA–CpG B nanovaccine for T-cell priming and initiating immune responses.

### Therapeutic and prophylactic efficacy of the pErNP–OVA–CpG B nanovaccine

**Fig. 3:**
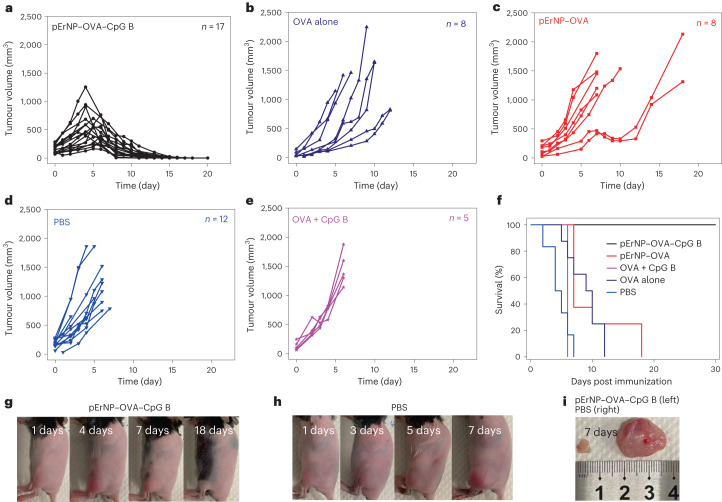
Therapeutic efficacy of nanovaccine for eradicating pre-existing tumours in mice. **a**–**e**, Tumour volume measured at different timepoints post s.c. injection of pErNP–OVA–CPG B (*n* = 17), OVA alone (*n* = 8), pErNP–OVA (*n* = 8), PBS (*n* = 8) and OVA mixed with CpG B (*n* = 5) respectively. *n* is the number of mice used for each group. **f**, Per cent survival graph of each animal groups treated in **a**–**e**. **g**,**h**, Photographs of E.G7–OVA tumour-bearing mice recorded on various days post tumour inoculation (−4 days) and treated on the same 0 day by pErNP–OVA–CpG B or PBS. **i**, Ex vivo photos of the solid tumours of pErNP–OVA–CpG B immunized and PBS-treated group taken on day 7. Source data

We investigated the therapeutic effects of subcutaneously injected pErNP–OVA–CpG B nanovaccine (*n* = 17) in E.G7 tumour-bearing mice, as well as pErNP–OVA (*n* = 8), OVA alone (*n* = 8), OVA mixed with CpG B (*n* = 5) and PBS only (*n* = 12). The first dose was administered 4 days after E.G7–OVA tumour cell inoculation on the left hindlimb, followed by boosting on days 7 and 14, except for the mice that had to be killed due to tumour burden. Tumour volumes ranged from ~35 mm^3^ to ~284 mm^3^ at the start of treatment (Fig. 3a–e). Mice treated with pErNP–OVA–CpG B exhibited the slowest tumour growth, with tumour volumes beginning to obviously shrink on approximately day 5 after the first dose. After the booster dose, tumours continued to shrink and eventually disappeared. Remarkably, all 17 mice treated with pErNP–OVA–CpG B survived with 100% efficacy (Fig. 3f,g and Supplementary Fig. 11) without any tumour regrowth monitored over ~30 days.

For mice treated by pErNP–OVA (*n* = 8), there was a discernible slowdown in tumour growth, especially for initially smaller tumours, after two doses of treatment (Fig. 3c). However, most tumours started to grow again unabated from day 11, even after the third dose. The pErNP–OVA treated group showed an improved survival rate of 25% observed over 14 days. The remaining treatment groups after two doses of PBS (*n* = 12), OVA (*n* = 8) alone and OVA + CpG B (*n* = 5) all showed rapid tumour growth with 0 % survival rate (Fig. 3f,h,i).

To evaluate the prophylactic potential of the pErNP–OVA–CpG B nanocomplex as a cancer vaccine, we subcutaneously injected E.G7–OVA cells into vaccinated healthy mice (*n* = 3) 1 week after the second dose of vaccine and compared them with healthy mice treated with two doses of PBS control (*n* = 3). No detectable solid tumours grew in mice immunized with two doses of pErNP–OVA–CpG B monitored for over 30 days (Supplementary Fig. 12), while the control group treated with two doses of PBS injection showed rapid tumour growth, reaching ~1,500 mm^3^ volume within 7 days following cancer cell inoculation (Supplementary Fig. 12). These results demonstrate that the pErNP–OVA–CpG B nanocomplex indeed induces strong protective antitumour immunity, making it an efficient prophylactic vaccine.

For E.G7 tumour-bearing mice, we noticed the pEr–OVA–CpG B vaccine signal appearing in the tumour 6 h post s.c. injection (Fig. 2). For comparison, we injected pErNP-P^3^ nanoparticles without OVA and CpG B conjugation into E.G-7 tumour-bearing mice (Supplementary Fig. 13, *n* = 3). Similar to the pErNP–OVA–CpG B nanovaccine, the pErNP-P^3^ particles also reached the iLN and tumour (Supplementary Fig. 13). We then collected blood samples from the mice and separated plasma from blood cells using ethylenediaminetetraacetic acid-treated plasma tubes. We observed that both pErNP–OVA–CpG B and pErNP-P^3^ nanoparticle-treated mice had strong pEr signals in their plasma and not in the cell pellets, suggesting that the pEr signal observed in the tumour originated from particles entering blood circulation (after s.c. injection) and then leaking into tumour through enhanced permeation and retention effect. This finding prompted us to perform another control experiment of injecting our pErNP–OVA–CpG B vaccine intravenously (instead of at tail base subcutaneously) into EG.7 tumour-bearing mice (*n* = 2). We did observe strong tumour uptake of the vaccine but not in the LNs, and tumours grew rapidly to reach the mortality limit without treatment effect. This suggested s.c. injection of our vaccine was superior to intravenous (i.v.) injection and strong immune responses were initiated in the lymph nodes, not in the tumour.

### In vivo dynamic NIR-II molecular imaging of CD8 and peptide–MHC-I tetramer targets

**Fig. 4:**
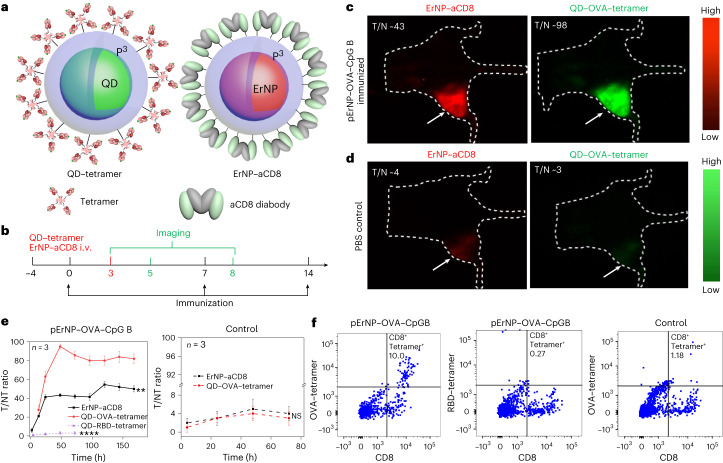
In vivo two-plex NIR-IIb molecular imaging of antigen-specific CD8^+^ T cells in wide-field mode. **a**, Schematics of QD functionalized by P^3^ coating and conjugated to biotinylated H-2K^b^ chicken ova 257–264 SIINFEKL, to form QD–OVA–tetramer (left) and ErNP functionalized with P^3^ coating and conjugated to CD8 diabody (right). **b**, Immunization and imaging schedule, C57BL/6 mice were immunized with the pErNP–OVA–CpG B nanovaccine or PBS on days 0, 7 and 14. On day 3, two probes (ErNP–aCD8 and QD–OVA–tetramer) were injected intravenously followed by imaging at various times, as indicated. **c**, Representative wide-field NIR-IIb molecular images from one of three mice bearing E.G-7 tumours immunized with pErNP–OVA–CpG B, recorded 48 h after i.v. injection of ErNP–aCD8 and QD–OVA–tetramer (for details, see Supplementary Fig. 14). Imaging conditions for ErNP–aCD8 channel: 940 nm excitation with a power density of ~50 mW cm^−2^, 1,500–1,700 nm detection, exposure times 20 ms, lifetime mode. QD: 860 nm excitation with a power density of ~50 mW cm^−2^, 1,500–1,700 nm detection, exposure times 50 ms, CW mode. **d**, The same as **c** for a mouse treated with PBS buffer as a control (for details, see Supplementary Fig. 15, *n* = 3). **e**, The tumour-to-normal tissue (T/NT) signal ratios of ErNP–aCD8 and QD–tetramer in tumour plotted as a function of time. T/NT was measured by ImageJ/Fiji using the ratio of fluorescence signals in the whole tumour area over the background without vasculature. For immunized group: ErNP–aCD8 versus QD–OVA–tetramer, *P* < 0.0001; QD–RBD–tetramer versus QD–OVA–tetramer, *P* = 0.0012; for control group: ErNP–aCD8 versus QD–OVA–tetramer, *P* = 0.4503. All data are from three independent experiments and are presented as mean ± standard deviation. Two-sided Student’s *t*-tests were used for the comparisons. **f**, Ex vivo FACS analysis of tumour-infiltrating CD8^+^ T cells from pErNP–OVA–CpG B vaccinated or control PBS-treated mice. Tumour-extracted cells from vaccinated animals were stained with OVA-specific SIINFEKL-H2K^b^ tetramer^+^ (left) or an irrelevant tetramer (RBD tetramer; middle). Cells from PBS-treated control animals were stained with the OVA-specific tetramer (right). The upper right quadrant of each plot shows the percentage of tetramer^+^ CD8^+^ T cells within the viable CD3^+^ T-cell population (for gating strategy, see Supplementary Fig. 22). Tumour cells are CD8^neg^CD4^+^CD3^+^ (Supplementary Fig. 26).

The ~100% therapeutic efficacy of eradicating pre-existing tumours in mice indicated strong adaptive immune responses elicited by the pErNP–OVA–CpG B nanocomplex. Since antigen-specific CD8^+^ CTLs are responsible for attacking and killing antigen-expressing cancer cells in tumours^34,35^, we explored spatially mapping/imaging and temporally tracking antigen-specific CTLs for assessing immune responses in vivo. We conjugated anti-CD8 diabody^36,37^ to our ErNP-P^3^ (second NIR-IIb emitting particle in Fig. 1) to form an ErNP–aCD8 probe (Fig. 4a right), and QDs (third NIR-IIb particle in Fig. 1) to tetramers of MHC class I H-2K(b) chicken ova 257–264 SIINFEKL peptide to afford QD–OVA–tetramer probes (Fig. 4a left; see Methods for conjugation details and Supplementary Fig. 14 for conjugation efficiency). In vivo, we administered pErNP–OVA–CpG B nanovaccine or control complexes subcutaneously, and then administered ErNP–aCD8 and QD–OVA–tetramer probes intravenously to target antigen-specific CD8^+^ CTLs residing in the tumour microenvironment through the blood. We employed the CW and lifetime modes multiplexed imaging of the probes to differentiate the three targets without signal crossing (Fig. 4b; for imaging details, see Methods, Table 1 and Supplementary Information).

For E.G7 tumour-bearing C57 BL/6 mice (*n* = 3) immunized on day 0, we intravenously injected a mixture of ErNP–aCD8 and QD–OVA–tetramer probes on day 3 and subsequently performed two-plex imaging at various timepoints (Fig. 4b). Wide-field whole-body NIR-IIb luminescence imaging showed weak signals in the spleen and negligible signal in the liver, heart and other organs for both animal groups (Fig. 4c,d). A strong signal in the E.G7–OVA tumour appeared in the CD8 channel (green, Fig. 4c,d and Supplementary Fig. 15 for *n* = 3 data) for mice immunized by the pErNP–OVA–CpG B vaccine, with the CD8 tumour-to-normal-tissue signal ratio (T/NT) reaching ~40 imaged on day 5 (48 h post injecting the probes, Fig. 4e). Meanwhile, we also observed a strong OVA–tetramer signal in the tumour (red, Fig. 4c and Supplementary Fig. 15), following a similar trend as the CD8 channel intensity with a peak T/NT ratio of ~95. These highly reproducible imaging results (Supplementary Fig. 15) indicated OVA antigen-specific CD8^+^ CTLs accumulation in the tumour in response to pErNP–OVA–CpG B vaccination, which correlated well with effective cancer cell killing by CTLs and tumour shrinking. In strong contrast, the PBS-treated animal group without vaccination showed negligible signals in both CD8 and OVA–tetramer channels in the tumour, indicating no OVA-specific CTL in the tumour and correlating with unabated tumour growth (Fig. 4d,e right and Supplementary Fig. 16 for *n* = 3 data).

**Extended Data Fig. 1:**
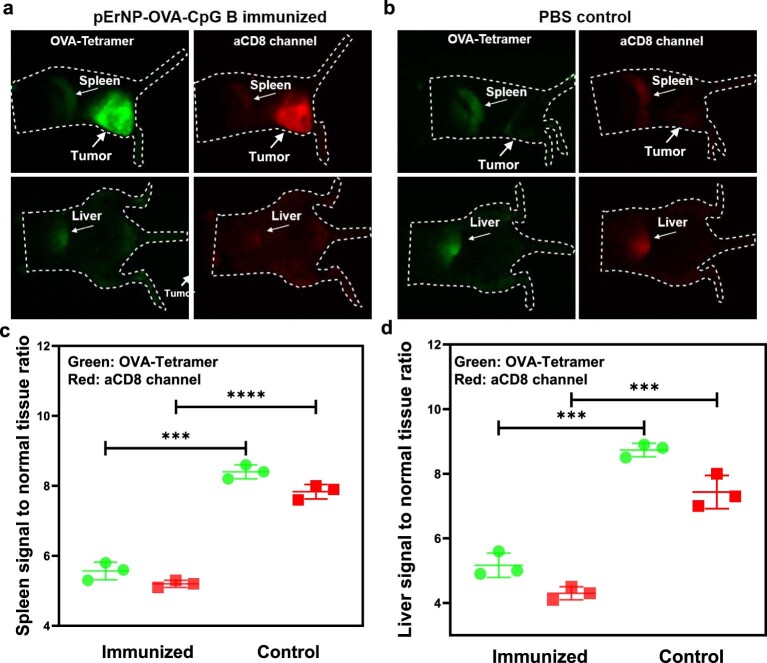
In vivo NIR-II molecular imaging of MHC-I OVA peptide and CD8 distribution in mice after immunization by nanovaccine vs. PBS control. **(a)** and **(b)** Wide-field NIR-IIb molecular images of mice bearing E.G7 tumors immunized with pErNP-OVA-CpG B and PBS, respectively, recorded 48 h after intravenous injection of ErNP-aCD8 and QD-OVA-Tetramer. **(a)** is the same mouse as Supplementary Fig. 14, Mouse 1. Here we show the images taken on the left and belly side to allow for comparisons. Imaging conditions for ErNP-aCD8 channel: 940 nm excitation with a power density of ~50 mW cm^−2^, 1500–1700 nm detection, exposure times 20 ms, lifetime mode. QD: 860 nm excitation with a power density of ~50 mW cm^−2^, 1500–1700 nm detection, exposure times 50 ms, CW mode. Note the strong signals in the tumor and weak liver/spleen signals in **(a)**, and the opposite in **(b)**. **(c)** Spleen-to-background signal ratios plotted to reveal the biodistribution of OVA-Tetramer (green, P = 0.0001) and ErNP-aCD8 (red, P < 0.0001) in mice bearing E.G-7 tumors immunized with pErNP-OVA-CpG B or PBS control. The data shows that with effective immunization, the spleen uptake of our probes was much lower than in PBS control mice, due to effective targeted tumor uptake of our probes seen in **(a)**. **(d)** Liver-to-background signal ratios plotted to reveal the biodistribution of OVA-Tetramer (green, P = 0.0001) and ErNP-aCD8 (red, P = 0.0006) in mice bearing E.G-7 tumors immunized with pErNP-OVA-CpG B or PBS (n = 3 in each group). The data shows that with effective immunization, the liver uptake of our probes was much lower than in PBS control mice, due to effective targeted tumor uptake of our probes seen in **(a)**. All data are from three independent experiments and are presented as means ± s.d. Two-sided Student’s t-tests were used for the comparisons.

The CD8 and tetramer signals inside the tumour in pErNP–OVA–CpG B immunized mice were much stronger than those in the spleen or liver (Extended Data Fig. 1a), suggesting extravasation from the blood circulation and specific targeting of our ErNP–CD8 and QD–tetramer probes to antigen-specific CTLs in the tumour microenvironment. For the PBS control group, we observed that the spleen and liver signals were stronger than in the tumour (Extended Data Fig. 1b), suggesting the lack of antigen-specific CTLs in the tumour and uptake of the probes by the reticuloendothelial system (including liver and spleen, Extended Data Fig. 1c,d). Admittedly, while our method was effective for molecular imaging in the tumour microenvironment for s.c. tumour models, it would not be effective for molecular imaging in the spleen or liver (such as in an orthotopic liver tumour model) due to reticuloendothelial system uptake of nanoparticles in general.

**Fig. 5:**
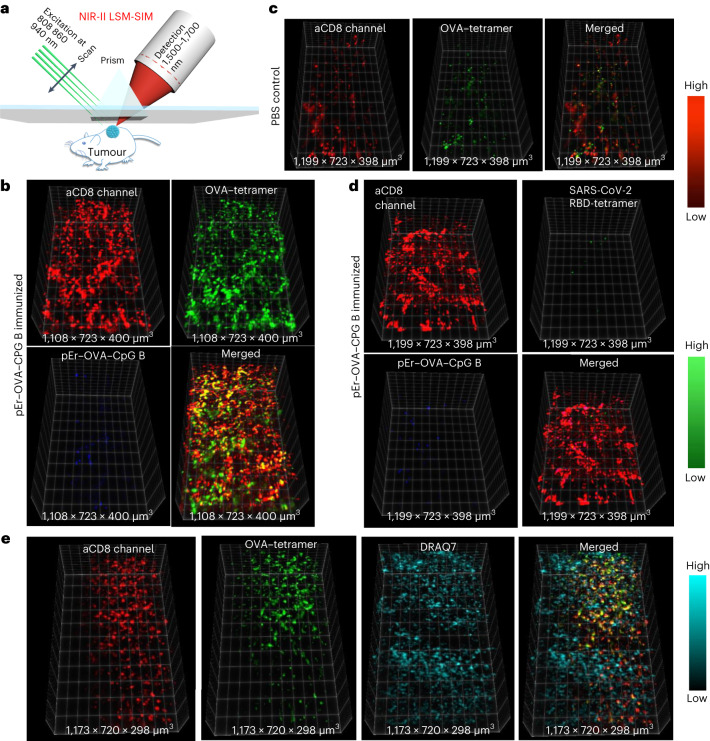
In vivo 3D volumetric NIR-II LSM-SIM imaging of antigen specific CD8^+^ CTLs in tumour microenvironment. **a**, Scheme of in vivo NIR-II LSM-SIM imaging^18^ with illumination and detection at 45° to the tumour (for details, see Supplementary Information). **b**, Three-dimensional volumetric NIR-II SIM images of ErNP–aCD8 (red), and QD–OVA–tetramer (green) recorded in the tumour in the pErNP–OVA–CpG B nanovaccine immunized mouse 48 h after i.v. injection of ErNP–aCD8, and QD–OVA–tetramer. It is important to note that the pEr signal is weak at this timepoint. **c**, The same as in **b** except that the mouse was ‘immunized’ by PBS buffer. **d**, The same as in **b** (mouse immunized with pErNP–OVA–CpG B nanovaccine) except that QD–RBD–tetramer was used instead of the QD–OVA–tetramer. **e**, Three-dimensional volumetric NIR-II SIM images of tumour tissue ex vivo. After in vivo SIM imaging, the mouse was killed under anaesthesia and the tumour was removed. The tumour was fixed in 10% neutral-buffered formalin for 30 min at room temperature, then washed three times with 1× PBS buffer and labelled with the nuclear dye, DRAQ7, for 3 h. After washing the tumour three times with 1× PBS buffer, it was preserved in glycerol at 4 °C for ex vivo imaging. Imaging conditions for ErNP: 940 nm excitation, 1,500–1,700 nm detection, exposure times 20 ms, lifetime mode; QD: 860 nm excitation, 1,500–1,700 nm detection, exposure times 100 ms, CW mode; pErNP: 808 nm excitation, 1,500–1,700 nm detection, exposure times 20 ms, lifetime mode; DRAQ7: 650 nm excitation, 690–850 nm detection, exposure times 100 ms, CW mode.

We also made an MHC-I tetramer negative control probe, by conjugating an irrelevant tetramer (peptide from the receptor binding domain (RBD) of severe acute respiratory syndrome coronavirus 2 (SARS-CoV-2) spike protein bound to H-2K^b^) to QDs and injecting the QD–RBD–tetramer into the E.G7 bearing mice immunized by pErNP–OVA–CpG B (Fig. 4e left and Supplementary Fig. 17). In this instance, although a strong CD8 signal was detected (Supplementary Fig. 17), a negligible QD–RBD–tetramer signal was observed in the OVA expressing tumour (Fig. 4e left dashed purple line, Fig. 5d and Supplementary Fig. 17), confirming the specificity of QD–OVA–tetramer for labelling OVA-specific CTLs in the tumour induced by the pErNP–OVA–CpG vaccination.

### In vivo one-photon 3D volumetric NIR-II LSM-SIM imaging of antigen-specific CD8^+^ CTLs in tumours

We recently developed a non-invasive in vivo NIR-II LSM-SIM with long excitation and emission wavelengths up to ~1,540 nm and ~1,700 nm, respectively^18,27^. This method suppressed light scattering and out-of-focus background, affording spatial resolution down to single-cell level at ~1 mm tissue depth in vivo. Our LSM-SIM imaging achieved a spatial resolution of approximately 1.7 ± 0.2 μm × 1.1 ± 0.2 μm × 1.6 ± 0.1 μm, making it suitable for resolving single cells in vivo as shown for CD4^+^ and OX40^+^ cells in the tumour microenvironment by intratumourally injected CpG (refs. ^18,27^). To image antigen-specific CD8^+^ CTLs with high resolution in the tumour microenvironment, we performed three-plex 3D volumetric LSM-SIM imaging on day 5 (48 h post i.v. injection of ErNP–aCD8 and QD–OVA–tetramer probes on day 3) post vaccination by pErNP–OVA–CpG B. While the pErNP vaccine signal in the tumour was weak by this time after the first vaccination, both ErNP–aCD8 (red colour in Fig. 5b) and QD–OVA–tetramer (green colour in Fig. 5b) signals were strong. Importantly, the CD8 and OVA–tetramer signals largely overlapped (yellow colour in Fig. 5b), corresponding to individual antigen- specific CTLs. In contrast, for the control mice ‘vaccinated’ by PBS buffer injection, the signal of CD8 and OVA–tetramer were both weak and lacked obvious signal overlap (Fig. 5c). For mice injected with ErNP–aCD8 and negative control QD–RBD–tetramer probes, strong CD8 signals were observed in the tumours without any obvious QD–RBD–tetramer signal (Fig. 5d). We then proceeded to resect the tumour and applied a NIR DRAQ7 nuclear dye to stain the cell nuclei^25^. Ex vivo SIM imaging of DRAQ7 revealed distinct labelling of the nuclei, without overlapping with either aCD8 or OVA–tetramer signals on the cell surfaces (as shown in Fig. 5e), but with feature sizes similar to those of aCD8 and OVA–tetramer signals. These results suggested NIR-IIb LSM-SIM is capable of imaging at the single-cell level within intact tumours in vivo, similar to previous results of CD4 and OX40 imaging^18,27^.

We have shown non-invasive in vivo imaging of antigen-specific CD8^+^ CTLs through intact tissue with cellular resolution using NIR-IIb imaging. To validate the in vivo imaging results, we used fluorophore-conjugated anti-mouse CD3 and CD8 antibodies, RBD–tetramer and OVA–tetramer to perform ex vivo flow cytometry analysis of cells extracted from tumours (Fig. 4f and Supplementary Figs. 18, 19, 22 and 23 used protein kinase inhibitor (PKI) dasatinib for optimized tetramer staining^38^; Supplementary Figs. 20, 21, 24 and 25 show cells without PKI). Indeed, flow cytometry detected substantially more OVA-specific CD8^+^ T cells in tumours from mice vaccinated with pErNP–OVA–CpG B than in tumours from control mice treated with PBS (Fig. 4f and Supplementary Figs. 18–21). For the irrelevant RBD–tetramer control group, ex vivo flow cytometry showed negligible staining of CD8^+^ T cells in tumours (Fig. 4f and Supplementary Figs. 18–21), consistent with the results of in vivo NIR-IIb imaging (Fig. 5d and Supplementary Fig. 17). This further confirmed NIR-II/SWIR molecular imaging as a promising approach for assessing immune responses in animal models in vivo.

## Discussion

The overarching goal of this work was to explore the potential of NIR-II/SWIR luminescent/fluorescent nanoparticles as vaccine delivery carriers as well as in vivo molecular imaging probes for investigating the immune responses of vaccinated mice. Our findings may guide the design of better vaccines and afford a deeper understanding of the immune systems at the cellular level by imaging and tracking longitudinally without killing the mice. Although many types of nanoparticle have been developed for vaccine and drug delivery^39–43^, we deployed nanoparticles luminescent in the ~1,500–1,700 nm NIR-IIb range, allowing for deep tissue in vivo imaging of vaccine trafficking by low-resolution and high-resolution modalities.

Since the first in vivo NIR-II imaging with carbon nanotubes in 2009 (ref. ^19^), many groups have demonstrated that in vivo NIR-II fluorescence imaging in the 1,000–3,000 nm range can offer superior imaging depth and signal-to-background ratios compared with traditional NIR imaging in the 800–900 nm range, owing to reduced light scattering and autofluorescence^18–23,27,30,44–46^. In particular, imaging performance was maximized by detecting in the NIR-IIb (1,500–1,700 nm) subwindow, which allowed in vivo imaging at the single-cell level at depths of millimetres^18,25,27^ and imaging guided surgical removal of cancer cells down to the few-cell level^26^. The current work added pErNPs to the ErNP and QD NIR-IIb probe set. These probes were luminescent in the same emission range but could be excited by different excitation wavelengths or exhibited different excited-states lifetimes. These optical characteristics were exploited for three-plex in vivo imaging in the 1,500–1,700 nm range in CW or lifetime modes (Table 1), expanding from our previous work of using ErNP and QD for two-plex molecular imaging of PD-L1 and CD8 in mice treated by anti-PD-L1 checkpoint blockade immunotherapy^30^. Nevertheless, it is necessary to further expand the NIR-II probe sets beyond the currently achieved three for much higher degrees of multiplexed in vivo imaging of the vast number of biomarkers of the immune system.

Cancer treatment and prevention by immunotherapy and vaccination/immunization are powerful ways to fight cancer and have been an important field of basic immunological research with landmark clinical successes^47^. With the E.G7–OVA mouse tumour model, a wide range of nanoparticle approaches have been investigated for vaccine development and mechanistic understanding of the complex immune responses^11,13,43,48,49^. Our current pErNP–OVA–CpG B nanovaccine exhibited bright NIR-II/SWIR fluorescence. In vivo imaging clearly demonstrated effective vaccine trafficking through the lymphatic system and homing to tumour draining lymph nodes. This was an important first step in triggering a cascade of immune reactions in the lymph node including antigen presentation, B-cell activation/expansion/antibody production, and T-cell priming and induction of CTLs for infiltrating the tumour and attacking cancer cells^50–53^.

For 17 treated mice our pErNP–OVA–CpG B nanovaccine eradicated OVA-expressing E.G-7 tumours up to ~284 mm^3^ in size and afforded 100% survival without tumour regrowth, monitored over 30 days. In contrast, 75% of the mice in the control groups (*n* = 33, free pEr nanoparticles, OVA mixed with CpG B, pErNP–OVA and PBS buffer) had to be killed within 7 days due to heavy tumour burden, with 0% survival beyond 30 days. Vaccination of healthy mice by pErNP–OVA–CpG B also prophylactically protected mice against tumour challenge. Such high treatment and immunization efficacy of pErNP–OVA–CpG B was superior to most vaccines with similar components (nano-carrier, protein and adjuvant) in the literature^6,42,54^. We attributed the high efficacy to the unique structure of cross-linked hydrophilic polymer coating over the inorganic pErNP core, OVA antigen covalently conjugated to the polymer layer and CpG B electrostatically complexed to the surface positive charges. In vivo NIR-IIb imaging showed that a fraction of the nanovaccine migrated to the lymph nodes within hours of administration (Fig. 2b), with the remaining vaccine gradually released from the s.c. injection site (Fig. 2b). This could be correlated to the rapid and sustained immune responses and therapeutic effects detected.

Importantly, our previous work demonstrated that the cross-linked P^3^ coating on nanoparticles imparted high biocompatibility to various types of nanoparticles without discernable toxic effects in vivo, enabling their excretion from the body within ~2 weeks via the biliary pathway^24^. This coating also allowed for highly specific targeting of CD8, PD-L1, CD4, OX40, CD169 and MECA-79 in vivo through antibody conjugation, with little non-specific binding^25,27,30^. Here the same P^3^ functionalization of the pEr nanoparticles facilitated the highly efficient loading of antigenic vaccine and CpG B. In a control experiment, we attempted to coat pErNPs with phospholipid–polyethylene glycol (DSPE–PEG–NH_2_, a widely used coating in nanomedicine field^55–57^), and the resulting water soluble pEr–DSPE–NH_2_ nanoparticles were conjugated to OVA and CpG B to form pEr–DSPE–OVA–CpG B using the same method as pEr-P^3^–OVA–Cp G B nanocomplex. NIR-IIb imaging found that the pEr–DSPE–OVA–CpG B vaccine exhibited little trafficking to lymph nodes, correlating with the lack of immunotherapeutic efficacy or slowdown in tumour growth (Supplementary Fig. 27). Hence, NIR-II imaging with mouse models could provide a useful approach to guide the development of vaccines.

**Extended Data Fig. 2:**
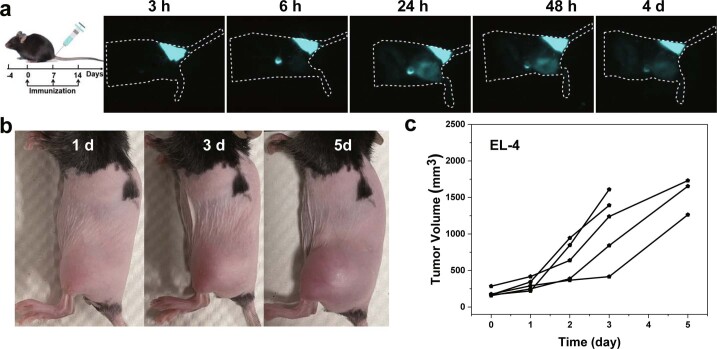
Treatment of mice bearing OVA-negative EL-4 tumors with the pEr-OVA-CpG B nanovaccine. **(a)** Immunization schedule, EL-4 tumor bearing C57BL/6 mice were immunized by subcutaneous injection at the mouse tail-base with the pErNP-OVA-CpG B nanovaccine on day 0, 7 and 14. Also shown are NIR-IIb luminescence images (975 nm excitation with a power density of ~50 mW cm^−2^, 1500–1700 nm detection, exposure times 20 ms, CW mode) showing the vaccine trafficking pathways after subcutaneous injection at the tail-base of a mouse. Images were recorded at different time points as indicated post injection. **(b)** Photographs of C57BL/6 mice treated with pErNP-OVA-CpG B vaccines at different time points. **(c)** Tumor volume measured at different time points post s.c. injection of pErNP-OVA-CPG B (n = 5). The EL-4 tumors showed no response to the pErNP-OVA-CpG B nanovaccine and grew unabated. Source data

Our pErNP–OVA nanocomplex vaccine slowed down tumour growth and extended mice survival (Fig. 3c). However, consistent with previous findings, we found that CpG B had a strong adjuvant effect, greatly boosting immune responses to the nanoparticle–OVA complex^58–60^ and resulting in a much more potent pErNP–OVA–CpG B vaccine (Fig. 3a). This effect was attributed to CpG binding to and activating APC^12^, inducing inflammatory cytokine production and enhancing antigen-specific immunity by shifting naive, inactive, OVA-specific CD8 T cells to cytotoxic, activated CD8 T cells^12,60^. Also, to confirm the antigen specificity of the immune response, we treated mice bearing OVA-negative (‘cold’) EL4 tumour with the pEr–OVA–CpG B vaccine. NIR-II imaging observed similar vaccine trafficking to mouse lymph nodes, but no therapeutic efficacy was observed, and the tumours grew unabated and rapidly (Extended Data Fig. 2).

It is widely accepted that immune activated, antigen-specific CD8^+^ CTLs are directly responsible for killing cancer cells^61^. Studies have shown that tumours with high levels of infiltrating CD8^+^ CTLs tend to have a better prognosis and respond more favourably to immunotherapies, such as immune checkpoint inhibitors^31,62^. This suggests that CD8^+^ CTLs play a key role in killing cancer cells and controlling tumour growth^31,38,62,63^. Moreover, recent research has highlighted the importance of the quality, as well as the quantity, of CD8^+^ CTLs in the tumour microenvironment. For example, CD8^+^ CTLs that are exhausted, are dysfunctional or have low functional avidity may be less effective at killing cancer cells^64–66^. Therefore, monitoring the function and quality of CD8^+^ CTLs within the tumour microenvironment may provide important information about the immune response to cancer and the potential efficacy of immunotherapies. Previous studies employed ex vivo flow cytometry to confirm antigen-specific CD8^+^ CTLs extracted from tumours stained by CD8^+^ antibody and fluorophore-conjugated peptide-MHC-I (p-MHC-I) tetramer. The p-MHC-I tetramer approach^62^ has empowered the investigation of cellular immune responses and the important roles of CD8^+^ CTLs. In the current work, we established in vivo imaging of antigen-specific CD8^+^ CTLs in the tumour microenvironment induced by the pErNP–OVA–CpG B nanovaccine targeted to the tumour draining lymph node, using two additional NIR-IIb emitting nanoparticles functionalized with the same P^3^ coating (as the pErNP vaccine carrier) and conjugated to CD8 diabody and pMHC-I OVA–tetramer, respectively (Fig. 4a). Intravenous injection of these probes afforded ~5.5 h of blood circulation times^30^, allowing extravasation of the probes into the tumour microenvironment to specifically recognize and bind to the CTLs. This was confirmed by in vivo wide-field and 3D volumetric microscopy imaging (Figs. 4c and 5) and validated by ex vivo flow cytometry analysis (Fig. 4f). These results established a method of in vivo molecular imaging and tracking of antigen-specific CD8^+^ CTLs with cellular resolution at millimetres depth inside a tumour, with the capability of longitudinal imaging/tracking.

The functionalization chemistry for NIR-II/SWIR-emitting nanoparticles and subsequent antibody or protein conjugation were crucial to the specificity of the in vivo molecular imaging using the resulting NIR-II probes, and the chemistry employed in the current work was developed over a multi-year period^24–27,30^. Nevertheless, much remains to be investigated and understood with the current cancer vaccine and mouse model. It is also highly desirable to perform multiplexed molecular imaging of immune cells in the lymph node in response to vaccination or immunotherapy, combined with imaging of the tumour microenvironment for a deeper understanding of cancer vaccines and immunotherapeutics.

## Outlook

We have developed a nanovaccine that can be tracked using the NIR-II/SWIR imaging modality, for pre-clinical research. Our experiments showed efficient trafficking of the nanovaccine through lymphatic vessels to tumour draining lymph nodes in mice, with migration to secondary lymph nodes also observed. High tumour luminescence of the vaccine carrier pErNP was observed, with mice exhibiting favourable immunotherapeutic effects. In tumour-bearing mice, the nanovaccine showed excellent tumour eradication and therapeutic efficacy, and prevented tumour growth in healthy mice. Furthermore, we employed NIR-II imaging by wide-field and light-sheet microscopy to visualize intratumoural antigen-specific CD8^+^ CTLs in vivo. All previous work performed ex vivo probing after killing mice. We monitored the recruitment of CD8^+^ CTLs to the tumour and longitudinally mapped out their distribution in the tumour microenvironment, which is important for understanding the immune response to cancer and for developing new immunotherapies. Overall, the imaging and tracking capabilities of NIR-II/SWIR luminescent/fluorescent nanoparticles, which allow for millimetres of penetration depth, high molecular specificity and non-invasive longitudinal monitoring, make them a promising class of vaccine delivery carriers for basic research involving the immune system at the cellular level and in vivo, and for designing better vaccines.

## Methods

### Synthesis of NIR PbS/CdS QD and surface modification with P^3^ coating

The synthesis of NIR PbS/CdS QD and their surface modification with a P^3^ coating were performed according to a recent publication, resulting in an emission peak at about 1,880 nm in PBS buffer^25^. Detailed information can be found in Supplementary Information.

### Synthesis of hexagonal β-phase pErNP

Synthesis of NaErF_4_ Core Nanocrystals. Hexagonal phase (β) NaErF_4_ nanocrystals were synthesized using the previously reported method with some modifications^31^. In a typical synthesis, erbium (III) acetate hydrate (0.258 g), oleic acid (4.8 ml) and 1-octadecene (12 ml) were mixed in a 50 ml flask and heated to 150 °C in an Ar flow for 30 min, then left to cool down to 50 °C. Afterwards, a methanol solution (10 ml) containing ammonium fluoride (4 mmol) and sodium hydroxide (2.5 mmol) was added and stirred for 1 h. The reaction mixture was gradually heated to 300 °C at a rate of approximately 10 °C min^−1^ under argon after removing the methanol by heating the solution to 100 °C. The solution was maintained at this temperature for 60 min and then raised to 305 °C for an additional 19 min. Finally, the solution was cooled to room temperature to obtain the core NaErF_4_ nanocrystals. The nanocrystals were collected by centrifugation (4,400 r.p.m. for 30 min) after adding ethanol and were then dispersed in cyclohexane. The detailed information is provided in Supplementary Information.

The core–shell nanoparticles of NaErF_4_/NaYF_4_ were synthesized using the epitaxial growth method to coat hexagonal β-phase NaYF_4_ nanocrystals onto the NaErF_4_ core nanocrystals. In brief, a mixture of sodium trifluoroacetate (0.136 g), yttrium trifluoroacetate (0.428 g), oleic acid (6.4 ml) and 1-octadecene (6.5 ml) was combined with the NaErF_4_ core nanocrystals synthesized in the previous step in 3 ml of cyclohexane. The solution was heated under vacuum to 120 °C for 30 min to remove the cyclohexane, and then the temperature was increased to 300 °C and kept for 75 min. The temperature was further increased to 305 °C and kept for 19 min, followed by allowing it to decrease to room temperature. The resulting nanoparticles were washed with ethanol and collected by centrifugation (4,400 r.p.m. for 30 min) three times, and then dispersed in 3 ml of cyclohexane. This procedure was repeated once to coat another shell, forming the final pErNPs.

### Synthesis of cubic α-phase ErNPs

ErNPs were synthesized according to a previously described method with slight modifications^30^. Synthesis of α-NaYbF_4_:Ce, Er, Zn (core) nanoparticles. In a typical synthesis, 0.075 mmol Zn(CH_3_COO)_2_ and 0.75 mmol RE(CH_3_COO)_3_ (RE: 96% Yb, 2% Ce, 2% Er) were mixed with oleic acid (15 mmol) and 1-Octadecene (ODE) (37.5 mmol) at room temperature. The solution was vigorously stirred and heated to 150 °C for 30 min under argon and then cooled to 50 °C. Next, NaOH (75 mg) and NH_4_F (111 mg) dissolved in methanol (8 ml) were added into the above solution and reacted for 1 h at 50 °C under argon. Further, the solution was heated to 100 °C and then the heating temperature was set to 295 °C for 1 h. Afterwards, the solution was heated to 300 °C for another 20 min before cooling down to room temperature. The final core nanocrystals were collected by centrifugation (4,400 r.p.m. for 30 min), washed with ethanol and dispersed in 3 ml cyclohexane for further coating. Note that the synthesis of α-NaYbF_4_:Ce, Er (without Zn doping) followed the exact same procedures, except no Zn(CH_3_COO)_2_ was used.

For synthesis of α-NaYbF_4_, Ce, Er, Zn/NaYF_4_ (core–shell) nanoparticles were used. Briefly, 1 mmol CF_3_COONa, 1 mmol Y(CF_3_COO)_3_ and the as-prepared core nanoparticles were mixed with oleic acid (20 mmol) and ODE (20 mmol) in a two-necked flask at room temperature. The solution was degassed for 30 min under vigorous stirring and then heated to 120 °C under vacuum for 30 min to remove water and oxygen. The solution was gradually heated to 300 °C for 75 min and then 305 °C for another 19 min under argon. After cooling down to room temperature, the resultant nanoparticles were centrifuged at 4,400 r.p.m. for 30 min, washed with ethanol, and dispersed in 3 ml cyclohexane (the mass concentration was ~80 mg ml^−1^). The above synthetic procedure was repeated once to form the final ErNPs.

### Surface modification of ErNPs or pErNPs with P^3^ coating

Poly(maleic anhydride-alt-1-octadecene) (PMH) (80 mg, 30–50 kDa) dissolved in 5 ml chloroform was mixed with pErNPs/ErNPs (32 mg) dispersed in cyclohexane. After stirring the solution for 1 h, the organic solvent was evaporated overnight. Then, an aqueous solution of 4-(dimethylamino)pyridine (DMAP) (80 mg in 6 ml water) was added, and the mixture was sonicated to obtain a well-dispersed solution of pErNPs/ErNPs. The solution was then centrifuged at 14,000 r.p.m. for 2 h to remove the excess PMH and DMAP, and the sediment was resuspended in 3 ml 2-(N-morpholino)ethanesulfonic acid (MES) solution (10 mM, pH 11). Then, 8Arm–PEG–NH_2_ (12 mg) dissolved in 3 ml MES solution and EDC (8 mg) was added to the solution and shaken for 3 h. The excess –COOH groups derived from PMH were quenched using Tris base (40 mg) and EDC (20 mg) dissolved in MES solution. After 3 h of reaction, large aggregates were removed by centrifugation at 4,400 r.p.m. for 30 min, and excess PEG and reaction byproducts were removed by dialysis against water for 12 h (using a 300 kDa membrane; water was changed more than eight times). The solution was then concentrated by centrifugal filter (100 kDa) once, and finally dispersed in 3 ml MES solution. Poly(acrylic acid) (PAA) (4 mg) and 1-(3-dimethylaminopropyl)-3-ethylcarbodiimide hydrochloride (EDC) (8 mg) dispersed in 3 ml MES solution were added to the above solution. After 1 h of reaction, potential large floccules were removed by centrifugation (4,400 r.p.m. for 30 min). The excess PAA was excluded by washing the supernatant using a centrifugal filter (100 kDa) and then dispersed in 3 ml MES solution. Finally, mPEG–NH_2_ (4 mg), 8Arm–PEG–NH_2_ (0.8 mg) and EDC (8 mg) dissolved in 3 ml MES solution were added for the final shell coating (P^3^ coating) and reacted for 3 h, yielding the final product, either ErNP-P^3^ or pErNP-P^3^.

### Synthesis of pErNP–OVA–CpG B and pErNP–OVA–CpG B-FITC nanovaccine complexes

pErNP-P^3^ (200 μl MES solution, containing ~2 mg pErNPs), OVA (100 μg in de-ionized water, 5 μg μl^−1^), EDC (1.5 mg) and 800 μl MES solution (10 mM, pH 11) were mixed together and shaken for 3 h. The solution was then centrifuged for 30 min with a speed of 4,400 r.p.m. to remove potential large floccules. The supernatant was washed by centrifugal filter (100 kDa) three times, and then dispersed in 100 μl 1× PBS solution to the former pEr–OVA solution. This pEr–OVA solution was mixed with CpG B (5 μg in de-ionized water, 1 μg μl^−1^) and incubated at room temperature for half an hour (for one injection).

The synthesis of pErNP–OVA–CpG B-FITC complex was the same as the above procedure, except CpG B-FITC was used instead of CpG B.

### Conjugation of aCD8 diabody on ErNP-P^3^

The conjugation of aCD8 to ErNP was prepared according to a previously described method^30^. Basically, ErNPs-P^3^ in MES solution (200 μl, containing ~2 mg ErNPs), anti-CD8α diabody (60 μg), EDC (1.5 mg) and 800 μl MES solution (10 mM, pH 11) were mixed together and shaken for 3 h. The solution was centrifuged for 30 min with a speed of 4,400 r.p.m. to remove potential large floccules. The supernatant was washed by centrifugal filter (100 kDa) three times, and then dispersed in 200 μl 1× PBS solution (for one injection).

### Conjugation of QD-P^3^ to streptavidin (SA) and to biotinylated MHC-I H-2K(b) chicken ova 257–264 SIINFEKL, to form QD–OVA–tetramer

The QD-P^3^–streptavidin was synthesized according to a previously described method^20^. QD-P^3^ (in 1× PBS buffer, 50 µl, (optical density) OD = 1), 50 µl SA (dissolved in 1× PBS, 1 mg ml^−1^, 53 kDa), 0.75 mg EDC dissolved in 50 µl MES buffer (pH 8.5) were added to 450 µl MES buffer (pH 8.5) and the reaction solution was shaken at room temperature for 3 h. After conjugation, the solution was centrifuged at 4,400 r.p.m. for 10 min, the precipitate was discarded and the supernatant was washed with 1× PBS buffer by 100 kDa centrifugal filter (12,000 r.p.m. for 30 min, four times). Finally, the QD-P^3^–streptavidin conjugates were dispersed in 100 µL 1x PBS buffer.

The QD-P^3^–SA conjugates and biotinylated MHC-I H-2K(b) chicken ova 257–264 SIINFEKL monomer (50 µl, 2 mg ml^−1^, 47,099 Da) were mixed and shaken at room temperature for 2 h and then washed three times with a 100 kDa filter to form QD–OVA–tetramer (each SA can bind to up to four biotinylated monomers).

### Characterization

NaErF_4_/NaYF_4_ (core–shell) nanoparticles were characterized by powder X-ray diffraction (Rigaku Miniflex 600 Benchtop) with Cu-Kα radiation. TEM images were taken with a FEI Tecnai G2 F20 X-TWIN Transmission Electron Microscope. DLS and zeta potential measurements were performed on a Malvern Zetasizer Nano ZS90. Fluorescence spectra of CpG B-FITC were taken with a Fluorolog-3 system (Horiba Jobin Yvon) using a charge coupled device detector. The luminescent properties of pErNP, ErNP and QD were studied using a home-built NIR spectrograph with a spectrometer (Acton SP2300i) equipped with a liquid-nitrogen-cooled InGaAs linear array detector (Princeton OMA-V). For pErNP and ErNP, a 975 nm diode laser was used as excitation, for QD, an 808 nm diode laser was used as excitation. Absorption spectra were acquired with a Cary 5000 ultraviolet-visible-NIR spectrophotometer (Varian) with a scan speed of 600 nm min^−1^.

Cryo-EM movie stacks were collected using a Glacios transmission electron microscope (Thermo Fisher Scientific) equipped with a K2 direct electron detector (Gatan) using the Serical-EM automation software^67^. The sample was prepared by applying 3 μl of a 0.5 mg ml^−1^ solution onto a glow-discharged (15 mA for 60 s) holey carbon grid (Quantifoil cupper R2/1 200 mesh) waiting for 10 s and blotting with filter papers for 6 s at force 5, 100% humidity and 16 °C. The grid was then flash-frozen in liquid ethane using an automated plunge-freezing device (Vitrobot, Thermo Fisher Scientific). The cryo-EM data were collected at a nominal magnification of 36,000× or 45,000× and a pixel size of ~1.2 or 0.93 Å with a defocus value of −3.0 or −5 μm. Each movie stack contained ~40 frames with a total electron does of ~50 e^−^ Å^−2^. The data were processed using a combination of MotionCor2, RELION, EMAN2 and ImageJ^68–71^.

The lifetime measurement was performed on an InGaAs photomultiplier tube (PMT, H12397-75, Hamamatsu) through a multimode fibre. Photoluminescence (PL) decay curves were fit with a typical bi-exponential function (*y* = *A*_1_ × exp(−*x*/*τ*_1_) + *A*_2_ × exp(−*x*/*τ*_2_) + *y*_0_). Average lifetime (*τ*) was calculated from two lifetime components, *τ*_1_ and *τ*_2_, by using the following equation:

$$\tau =({A}_{1}{\tau }_{1}^{2}+{A}_{2}{\tau }_{2}^{2})/({A}_{1}{\tau }_{1}+{A}_{2}{\tau }_{2})$$

where *A*_1_ and *A*_2_ represent the relative amplitude of *τ*_1_ and *τ*_2_, respectively, and were obtained from the fitting of the bi-exponential function.

### In vivo wide-field NIR-II fluorescence imaging in CW and lifetime modes

The NIR-II wide-field fluorescence images were acquired using a 2D water-cooled InGaAs camera (Ninox640, Raptor Photonics) operating at −21 °C, with a 1,500 nm long-pass filter (FELH1,500, Thorlabs) to generate imaging windows in the range of ~1,500–1,700 nm (within the InGaAs camera detection range, below ~1,700 nm). For three-plex imaging, QD–OVA–tetramer was excited by an 860 nm laser with a power density of ~50 mW cm^−2^, using a 1,500 nm long-pass filter, exposure times 50 ms, CW model. The fluorescence of QD–OVA–tetramer was collected in the range of 1,500–1,700 nm.

To distinguish the luminescence of pErNPs and ErNPs (with long-lived ~1,550 nm luminescence of 2.7 ms and 7.1 ms, respectively) from the short-lived fluorescence of QDs in the same 1,500–1,700 nm NIR-IIb window, a time-resolved imaging technique was developed. The signals from pErNPs and ErNPs were collected using a pulsed laser for excitation and a delay time set by computer control to allow full fading of the short-lived fluorescence of QDs before recording. Laser excitation was changed for lifetime imaging to distinguish between pErNPs and ErNPs. Specifically, pErNPs were imaged by detecting emission in the range of 1,500–1,700 nm with a 20 ms exposure time, 1 ms after turning off an 808 nm pulsed laser (illumination duration 1 ms) at a power density of ~70 mW cm^−2^. The 808 nm laser excitation of pErNPs did not generate emission from ErNPs, which absorb in the range of ~910–1,000 nm. Similarly, for imaging ErNPs, emission in the range of 1,500–1,700 nm was detected with a 20 ms exposure time, 1 ms after turning off a 940 nm pulsed laser (illumination duration 1 ms) at a power density of ~50 mW cm^−2^. The 940 nm laser excitation of ErNPs did not generate emission from pErNPs.

The imaging scheme described above (QD imaging in CW mode with 860 nm excitation; pErNP imaging in lifetime mode with 808 nm excitation; ErNP imaging in lifetime mode with 940 nm excitation) enabled imaging of the three probes one at a time without signal crossover, allowing multiplexed molecular imaging after the probes were conjugated to different antibodies or antigens.

### Data processing

The raw data of luminescent and absorbance spectra were processed in Origin 2021 (OriginLab). PL decay curves were fit with Origin 2021 as well. The pErNP signal of wide-field NIR-II fluorescence imaging in tumour or iLN was chosen in the whole tumour or the whole iLN area, the background was measured from a randomly selected area without vasculature or lymphatic vessel. The NIR-II LSM-SIM 3D images were constructed in ImageJ/Fiji (2021) by using the function of affine transform. The merged picture of muti-colour images was also performed in ImageJ/Fiji. Statistical comparisons between two groups were determined by two-tailed Student’s *t*-test. Statistical analysis was performed using GraphPad Prism 8.0. For statistical analysis, *P* < 0.05 was considered statistically significant: *P < 0.05, **P < 0.01, ***P < 0.001 and ****P < 0.0001.

### Mouse handling

All animal experiments conducted in this study were approved by Stanford Institutional Animal Care and Use Committee. All procedures adhered strictly to the guidelines set forth in the National Institutes of Health Guide for the Care and Use of Laboratory Animals. The mice were housed on a 12 h light/dark cycle at room temperature 20–25 °C and humidity 50–65% in Veterinary Service Center at Stanford University. The Stanford Veterinary Service Center facility supplied the bedding, nesting material, food and water for animals. For wide-field NIR-II fluorescence imaging in tumour or iLN, the pErNP signal was selected from the entire tumour or iLN area, while the background was measured from a randomly selected area without vasculature or lymphatic vessels. NIR-II LSM-SIM 3D images were constructed in ImageJ/Fiji (2021) using the affine transform function, and the merged picture of multi-colour images was also performed in ImageJ/Fiji.

### Cell culture

E.G7–OVA cells were obtained from the American Type Culture Collection (ATCC) and cultured in RPMI 1640 medium, which was supplemented with 2 mM l-glutamine, 1.5 g l^−1^ sodium bicarbonate, 4.5 g l^−1^ glucose, 10 mM HEPES and 1.0 mM sodium pyruvate. The medium was also supplemented with 0.05 mM 2-mercaptoethanol, 0.4 mg ml^−1^ G418 (90%), and 10% foetal bovine serum (FBS), as recommended by ATCC. We performed flow cytometry analysis to determine the expression of CD3, CD4 and CD8 on the EG-7 OVA tumour cells. The EG-7 OVA tumour cells were negative for CD8 and positive for CD3 and CD4 expression, indicating that they are CD4^+^ T lymphoma cells (Supplementary Fig. 26). EL4 cells were also obtained from ATCC and cultured in RPMI 1640 medium supplemented with 10% FBS.

### Administration of various formulations of vaccine in tumour-bearing mice

C57BL/6 mice (6–7 weeks, 15–20 g) were randomly divided into five groups (*n* = 5–17 per group) and inoculated with E.G7–OVA or EL 4 cells (2 × 10^6^ to 5 × 10^6^) in the left hindlimb. Tumours were allowed to grow to 50–300 mm^3^. Four days later, mice were immunized subcutaneously at the base of the tail with 100 μl 1× PBS solution of one of the following formulations: OVA (100 μg per mouse), pEr-OVA (100 μg OVA and 2 mg pErNP per mouse), OVA + CpG B (100 μg OVA and 5 μg CpG B per mouse) and pEr-OVA-CpG B (100 μg OVA, 5 μg CpG B and 2 mg pErNP per mouse). Note that ~95.6% OVA was actually conjugated to pErNP (see ‘Conjugation efficiencies of various biomolecules on nanoparticles’ in Supplementary Information). The control groups were treated with 100 μl of 1× PBS. Mice were immunized at days 0, 7 and 14. Tumours were measured with a digital caliper, and tumour volumes were calculated using the formula: *L* × *W*^2^/2, where *L* is the length and *W* is the width of the tumour.

### Tumour challenge

C57BL/6 mice (6–7 weeks, 15–20 g) were subcutaneously immunized with either 100 μl of 1× PBS (control group, *n* = 3) or 100 μl of 1× PBS containing pEr–OVA–CpG B (100 μg OVA, 5 μg CpG B and 2 mg pErNP per mouse, *n* = 3) at the base of the tail on days 0, 7 and 14. Seven days after the last immunization, mice were injected subcutaneously with 1 × 10^7^ E.G7–OVA cells into the left hindlimb.

### In vivo NIR-II LSM-SIM imaging

To map the antigen-specific CD8^+^ CTLs in tumours, we performed 3D NIR-II LSM-SIM imaging by using our home-built LSM-SIM^18^ (Fig. 5). This microscopy was equipped with a water-cooled InGaAs camera (Ninox640, Raptor) for orthogonally fluorescence detection in 400 nm to 1,700 nm wavelength range and multiple switchable lasers (808 nm, 940 nm, 975 nm and 1319 nm). Three days after pErNP–OVA–CpG B or PBS immunization, ErNP–aCD8 and QD–tetramer (QD–OVA–tetramer or QD–RBD–tetramer) were intravenously injected into the C57BL/6 mouse through the tail vein. Two days later, NIR-II LSM-SIM was performed to profile the 3D spatial distribution of ErNP–aCD8, pErNP–OVA–CpG B and QD–tetramer in the E.G7 tumour. As shown in Fig. 5a, the illumination and the detection objective were arranged 45° to the E.G7 tumour.

Fluorescence images were recorded at each optical section using Labview software to synchronize the Galvo mirror, motorized stage and camera through a data acquisition card (NI USB-6210). The scanning step was 4 μm along the *x* direction, and one step movement required 100 ms confined by the stage (M-VP-25XL, Newport). Excitation and emission collection were performed using a 5× objective (numerical aperture 0.12, Leica N Plan) and a 10× objective (numerical aperture 0.25, Olympus ULWD MIRPlan), respectively. The period of structured illumination was 2.6–3.6 times the full width at half maximum of the Gaussian excitation beam, consisting of five harmonics in the frequency domain for our five-phase reconstruction. The period was adjusted by the input voltage value for the Galvo mirror.

### Cell sorting of lymph node immune cells

Twenty-four hours after vaccination with pErNP–OVA–CpG B, the iLNs were collected, mechanically dissociated in media and filtered to obtain a single-cell suspension, followed by ammonium–chloride–potassium lysis to remove red blood cells. After centrifugation and resuspension in PBS/1% FBS, the cells were incubated with a mixture of anti-mouse CD3–AF488 (pan T cells), anti-mouse CD19–PE (B cells), anti-mouse CD11c–PE/Cy7 (DCs), anti-mouse F4/80–BV421 (macrophages), anti-mouse CD16/32 for Fc receptor blockade and live/dead dye. After a single wash and resuspension in PBS/1% FBS/2 mM ethylenediaminetetraacetic acid, the cells were filtered to remove cell aggregates and sorted into viable lineage marker^+^ populations, using a BD FACS Aria II sorter. See Supplementary Information for detailed methods and Supplementary Fig. 8 for sorting strategy.

### Flow cytometry analysis of antigen-specific CD8^+^ CTLs

Five days after immunization with pEr–OVA–CpG B nanovaccine (*n* = 3) or PBS buffer (*n* = 2), tumours were dissected, digested by DNAase and collagenase/hyaluronidase and filtered to obtain single-cell suspensions. The leukocyte fraction was purified using a mouse CD45-positive selection kit, treated with ammonium–chloride–potassium lysis to remove red blood cells, resuspended at 1 × 10^7^ cells ml^−1^ in media and recovered by incubation at 37 °C for 30 min with or without 50 nM PKI dasatinib to optimize tetramer staining^38^. To determine the frequency of OVA-specific CD8^+^ T cells, triplicate samples of leukocytes from each tumour were stained with an OVA peptide tetramer (SIINFEKL–H-2K^b^–PE) (ref. ^63^), or an irrelevant tetramer control, SARS-CoV-2 spike RBD peptide tetramer (VNFNFNGL–H-2K^b^–PE) or without tetramer, each followed by surface staining with anti-CD3–FITC and anti-CD8a–APC. Flow cytometry data were acquired using a BD LSRFortessa flow cytometer and analysed with FlowJo software v.10.8.1. See Supplementary Information for detailed methods and Supplementary Figs. 22 and 24 for gating strategy.

### Reporting summary

Further information on research design is available in the Nature Portfolio Reporting Summary linked to this article.

### Supplementary information

Supplementary Information Supplementary Methods, Figs. 1–27, Tables 1 and 2 and References.

Reporting Summary

Supplementary Video 1 pErNP–OVA–CpG B vaccine trafficking through lymphatic vessels between the injection site, iLN and aLN.

Supplementary Video 2 Movement of the vaccine in the lymphatic vessels trafficking to the iLN, captured by NIR-LSM imaging.

### Source data

Source Data for Fig. 3 and Extended Data Fig. 2 Source data for tumour growth.

## Data Availability

The main data supporting the results in this study are available within the paper and its Supplementary Information. The raw and analysed datasets generated during the study are too large to be publicly shared, yet they are available for research purposes from the corresponding author on reasonable request. Source data are provided with this paper for Fig. 3a–e and Extended Data Fig. 2c.
