# Supplementary material for: Shortwave-infrared-light-emitting probes for the in vivo tracking of cancer vaccines and the elicited immune responses
Source: Nat Biomed Eng. 2023 Aug 24;8(6):726–39. doi: 10.1038/s41551-023-01083-5 (PMC11250370; doi:10.1038/s41551-023-01083-5)
Supplement: Supplementary file 1 — Supplementary Methods, Figs. 1–27, Tables 1 and 2 and References. [file 41551_2023_1083_MOESM1_ESM.pdf]

nature biomedical engineering

Article

https://doi.org/10.1038/s41551-023-01083-5

Shortwave-infrared-light-emitting probes for the in vivo tracking of cancer vaccines and the elicited immune responses

In the format provided by the authors and unedited

Contents

Materials and methods
Synthesis of NIR PbS/CdS QD
Surface modification of PbS/CdS QD with P^3 coating
Conjugation efficiencies of various biomolecules on nanoparticles
Flow cytometry analysis of antigen-specific $CD8^+$ cells in tumour
Cell sorting of lymph node immune cells
Flow cytometry analysis of E.G7-OVA cell line
References

Materials and methods
Lead (II) chloride ($PbCl_2$), 1-Octadecene (ODE), cadmium oxide (CdO) and sulfur powder (sublimed) purchased from Alfa Aesar. Oleylamine, oleic acid, poly(maleic anhydride-alt-1-octadecene) (PMH; average molecular weight: 30-50 kDa), 2-(N-morpholino)ethanesulfonic acid (MES), 4-(dimethylamino)pyridine (DMAP), poly(acrylic acid) (PAA; average molecular weight: 1800 Da), 1-(3-dimethylaminopropyl)-3-ethylcarbodiimide hydrochloride (EDC), and 2-Amino-2-(hydroxymethyl)-1,3-propanediol (tris-base), rare-earth(III) acetate hydrate (RE: Yb, Er, Ce), zinc acetate, yttrium trifluoroacetate, sodium trifluoroacetate were purchased from Sigma-Aldrich. Hexane, toluene, chloroform, propidium iodide dye (P1304MP), and DI water were purchased from Fisher Scientific. Methoxy polyethylene glycol amine (mPEG-NH_2; average molecular weight: 5 kDa) was purchased from Laysan-Bio. 8-arm polyethylene glycol amine (8Arm-PEG-NH_2·HCl; average molecular weight: 40 kDa) was purchased from Advanced Biochemicals. All the chemicals were used without further purification. Collagenase/hyaluronidase (07912), and mouse TIL CD45 positive selection kit (100-0350) were purchased from StemCell Inc. CD8α-FITC (11-0081-82), and Live/Dead Fixable Near IR reagent (L10119) were purchased from Invitrogen. Class B CpG oligonucleotide - Murine TLR9 agonist and ovalbumin were purchased from InvivoGen. CD3-PE (100206), CD4-APC (100412), CD3-FITC (100204) and CD8α -APC (100712) were purchased from BioLegend. Biotinylated H-2K^b chicken ova monomer 257-264 SIINFEKL, biotinylated H-2K^b SARS-CoV-2 S 539-546 VNFNFNGL monomer, and PE-conjugated H-2K^b-SIINFEKL tetramer were provided by the NIH Tetramer Core Facility at Emory University. Anti-CD8 cys-diabody was provided by the lab of Anna M. Wu.

Synthesis of NIR PbS/CdS QD
The synthesis of NIR PbS/CdS quantum dot (QD) was carried out in accordance with a recently published protocol, resulting in an emission peak at about 1880 nm in PBS buffer [1].

Synthesis of PbS core QD. Briefly, 0.08 g of sulfur powder and 7.5 ml of oleylamine were added to a two-neck flask and heated at 120 °C under argon for 30 min.

In a separate three-neck flask, 1.668 g of $PbCl_2$ and 15 ml of oleylamine were mixed and degassed for 30 min at 120 °C before being heated to 165 °C under argon and held at this temperature for 15 min. Next, 4.5 ml of the sulfur precursor solution was injected into the Pb precursor solution while stirring and the temperature was maintained at 165 °C throughout the reaction.

After 180 min, the reaction was quenched by adding 20 ml of cold hexane and 30 ml ethanol, followed by centrifugation and re-suspension of the products in a mixture of hexane and oleic acid (at a 1: 2 volume ratio). This procedure was repeated three times, and the final product was re-suspended in 20 ml toluene after centrifugation.

Synthesis of PbS/CdS core/shell quantum dots. The mixture of CdO (1.2 g), oleic acid (8 ml), and ODE (20 ml) in a three-neck flask was heated to 200 °C for 1.5 hours under argon. Afterwards, the solution was cooled down to 100 °C and degassed under vacuum for 30 min. 5 ml toluene solution of the as-prepared PbS QDs bubbled with argon for 10 min was injected into the Cd precursor solution. The growth reaction was performed at 100 °C for 60 min and quench with 5 ml cold hexane. The PbS/CdS quantum dots were precipitated with ethanol and then re-dispersed in hexane.

Surface modification of PbS/CdS QD with P^3 coating
10 mg PMH dissolved in 0.5 ml chloroform was mixed with 2 mg QD dispersed in 0.5 ml cyclohexane and stirred overnight. The organic solvent was evaporated by rotary evaporator. 1 ml DMAP (10 mg) aqueous solution was added to the flask and then placed in a sonication bath to transfer the QD into the water phase. After precipitation of the QD by centrifugation (50000 r.p.m for 2.5 hours), the precipitate was resuspended in 0.5 ml MES (10 mM, pH = 8.5). 1.5 mg 8Arm-PEG-NH_2·HCl (MW 40 kDa) in 1 ml MES (pH=8.5) and 1 mg EDC were added and the solution was allowed to react for 3 hours on orbital shaker. The unreacted carboxylic groups were quenched with 5 mg Tris-base and 2.5 mg EDC. The purification of the product was performed by dialyzing the crude sample against water for 12 hours (300 kDa) and then centrifuging with a filter tube (100 kDa). The concentrated sample was dispersed in 0.5 ml MES (10 mM, pH = 8.5) solution followed by the addition of 0.5 mg PAA (MW 1800 Da) in 1 ml MES (10 mM, pH = 8.5) and 1 mg EDC. After 1h of reaction, the possible large floccules were removed by the centrifugation (4400 r.p.m for 30 min) and the supernatant was continuously washed by a centrifuge tube (100 kDa). For the final layer coating, the QD@PMH-8ArmPEG-PAA were mixed with 0.5 mg mPEG-NH_2 (MW 5 kDa), 0.1 mg 8Arm-PEG-NH_2·HCl (MW 40kDa) in 1 ml MES solution (10 mM, pH = 8.5) and 1 mg EDC, and the solution was allowed to react for 3 hours. Then, 5 mg Tris-base and 2.5 mg EDC were added to quench the reaction for 3 hours. The final

product was centrifuged at 4400 r.p.m for 30 min to remove potential large aggregates and the supernatant was washed by a centrifuge filter (100 kDa) for 4 times. The QD@PMH-8ArmPEG-PAA-mixed PEG (QD-P^3) were dispersed in 500 µl 1xPBS solution for further use.

Conjugation efficiencies of various biomolecules on nanoparticles
The conjugation efficiencies of OVA and aCD8 to their respective nanoparticles were determined by using Pierce BCA protein assay kit.

The Pierce BCA protein assay kit is a colorimetric assay that is commonly used to measure the total protein concentration in a sample[2,3]. In the BCA assay, the copper (II) ions in the reagent are reduced to copper (I) ions by the protein present in the sample[4]. The copper (I) ions form a colored complex with bicinchoninic acid, resulting in a change in absorbance that can be measured spectrophotometrically[4]. The wavelength at which the absorbance is measured in the BCA assay is typically around 562 nm. This specific wavelength corresponds to the peak absorbance of the colored complex formed between the protein and the BCA reagent. To use the kit for determining conjugation efficiencies, we first prepare several known concentrations of OVA and aCD8 solution, measure their absorbance at 562 nm, and generate a calibration curve (Supplementary Fig. 6). The calibration curve allows us to correlate the protein concentrations with the absorbance values obtained from the BCA assay. This enables us to accurately determine the protein concentration in our samples and calculate the conjugation efficiencies.

For the OVA conjugation, we used 2 mg pErNPs and 100 μg OVA. After conjugation, the solution was filtered through a 100 kDa filter and the filtrate was collected. The filtrate was then added to a 96 well plate, followed by the addition of the BCA reagent and incubation at 60 °C for 30 minutes. The absorbance of the filtrate at 562 nm was measured using a NanoDrop, resulting in an absorbance of 0.42, corresponding to a concentration of 4.4 mg/ml and a final conjugation efficiency of approximately 95.6%. For the anti-CD8α diabody conjugation, we used 2 mg ErNP and 60 µg anti-CD8α diabody. After conjugation and filtration, the absorbance of the filtrate at 562 nm was measured using the same method, resulting in an absorbance of 0.35, corresponding to a concentration of 3.3 mg/ml and a final conjugation efficiency of approximately 94.5%.

To assess the efficiency of the CpG B and pErNP-OVA binding, we used FITC-labeled CpG B instead of unlabeled CpG B. We mixed FITC-CpG B with pErNP-OVA, measured the FITC fluorescence intensity, and compared it to the fluorescence intensity after washing. We found that ~ 68% of the FITC-CpG B remained bound to pErNP-OVA even after rigorous washing with a 100 kDa filter at 4400 rpm for four cycles. It is worth noting that we did not perform this washing step before injection, which suggests that more CpG B may have been delivered than was measured in our assay. After a 24-hour storage period, we repeated the washing step and found that 41% of the FITC-CpG B remained bound to pErNP-OVA (Supplementary Fig. 7).

UV-vis absorption spectroscopy was used to quantify the tetramer/monomer conjugation efficiencies. We used a UV-vis spectrophotometer to obtain the absorbance spectrum of the monomer before conjugation. After conjugation, the solution was filtered using a 100 kDa filter and the filtrate was collected and concentrated to the same volume as the monomer. The difference in the absorbance spectrum between the monomer and sample allowed us to calculate the concentration of unreacted monomer in the sample. A tetramer conjugation efficiency of ~ 86% was obtained (Supplementary Fig. 13).

Flow cytometry analysis of antigen-specific $CD8^+$ cells in tumour
Five days after immunization with pEr-OVA-CpG B nanovaccine or PBS treatment as a control, tumours were harvested, cut into ~2 mm^3 pieces on ice under sterile conditions and digested with collagenase/hyaluronidase (STEMCELL Technologies, cat# 07912) and DNase I, using (per gram) 5ml solution containing 500 μl collagenase/hyaluronidase solution, and 750 μl DNase I solution. (STEMCELL Technologies, cat# 07900) in 3.75 ml RPMI 1640 medium for 25 minutes at 37 °C on a shaking platform, following manufacturer's protocol. The digested tissue was passed through a 70-µm cell strainer to obtain a single-cell suspension. The leukocyte-enriched fraction from tumour cells and infiltrating leukocytes was then isolated using the EasySep™ mouse CD45 positive selection kit (STEMCELL Technologies, cat#100-0350). The cells were pelleted via centrifugation at 300 g for 10 min at room temperature with the brake on low, then treated with ACK (ammonium-chloride-potassium) lysing buffer (Gibco, cat#A1049201) at room temperature for 5 min to lyse red blood cells, followed by centrifugation at 300 g for 10 min at room temperature with the brake on low. Cells were resuspended at 1×10^7 cells/ml in complete RPMI media (supplemented with 10% heat-inactivated FBS, 2 mM glutamine and 1% penicillin/streptomycin), seeded as

1×10^6 cells per well in a V-bottom 96-well plate in triplicate for each staining condition below, and recovered via incubation at 37 °C with 5% CO_2 with or without protein kinase inhibitor (PKI, 50 nM dasatinib)[5,6].

Leukocytes were stained with a panel of antibodies to detect CD3$^+$CD8$^+$ T cells and H-2K^b-SIINFEKL tetramer-positive cells; H-2K^b-VNFNFNGL-SARS-CoV-2 spike RBD peptide tetramers served as an irrelevant tetramer control and staining without tetramer was also done. Triplicate wells for each tumour/staining condition were incubated at 37 °C for 30 minutes with the H-2K^b-SIINFEKL tetramer (PE-conjugated, 1:200 dilution; NIH Tetramer Core Facility at Emory University), the H-2K^b-VNFNFNGL-RBD tetramer control (PE-conjugated, 1:213 dilution, NIH Tetramer Core Facility at Emory University). After tetramer staining, cells were incubated on ice for 20 minutes in the dark with Live/Dead Fixable Near IR reagent (1:1000, Invitrogen cat# L10119), anti-mouse CD3 (pan T cell marker: FITC-conjugated, 1:100 dilution; BioLegend) and anti-mouse CD8a (co-receptor on cytolytic T cells; BV421-conjugated, 1:100 dilution; BioLegend) and Fc block (rat anti-mouse CD16/CD32 Fc receptors; Becton Dickinson, cat#553142), followed by three washes with chilled PBS+10% heat-inactivated (HI) FBS and resuspended in 200 μl FACS buffer per well for analysis.

Flow cytometry data were acquired using a BD LSRFortessa flow cytometer (Becton Dickinson Biosciences) and analyzed using FlowJo software (version 10.8.1). 2x10^5 events were acquired at the initial stage of forward vs side scatter, followed by sequential gating as described in Supplementary Figs. 22 and 24. The frequency of antigen-specific CD8$^+$ T cell population was quantified as the percentage of tetramer+CD8+ events among viable CD3$^+$ T cells from infiltrating T cells and E.G7-OVA tumour cells (CD3$^+$, CD4$^+$, CD8^{neg}; Supplementary Fig. 26). The staining and flow cytometry experiments were done in triplicate for each tumour sample, under each staining condition.

Cell sorting of lymph node immune cells

24 hours after vaccination with pErNP-OVA-CpG B, the inguinal lymph nodes (iLNs) were harvested and mechanically dissociated using a syringe rubber head in complete RPMI media supplemented with 10% HI FBS, 2 mM glutamine, and 1% penicillin/streptomycin containing 25mM HEPES. The tissue suspension was passed through a 70 µm cell strainer to obtain a single-cell suspension, followed by ACK lysis to remove red blood cells. The cells were washed once by centrifugation at 300 × g for 5 minutes at 4 °C and resuspended in cold PBS with 1% FBS for subsequent staining. For surface staining, the cells were incubated with an antibody mix containing anti-mouse CD3-AF488 (pan T cells), anti-mouse CD19-PE (B cells), anti-mouse CD11c-PE/Cy7 (dendritic cells), anti-mouse F4/80-BV421 (macrophages), TruStain FcX™ PLUS (anti-mouse CD16/32 for Fc receptor blockade) [all BioLegend] and Live/Dead® Fixable Near IR dye (Thermo Fisher) for 15 minutes at 4 °C in dark. After incubation, cells were washed with cold PBS containing 1% FBS, then resuspended in cold PBS containing 1% FBS and 2 mM EDTA for sorting Cells were filtered with 70 um strainers right before sorting to remove clumps and cell aggregates. The fully stained cells were sorted into lineage marker+ populations at 4 °C using a BD FACS Aria II sorter. Compensation was set up using unstained and single color-stained cells.

Flow cytometry analysis of E.G7 cell line

For flow cytometry analysis, an E.G7 cell suspension was centrifuged at 400 × g for 5 minutes at 4 °C. The supernatant was then discarded, and the cell pellet was resuspended in staining buffer (PBS with 0.5% BSA) at a concentration of 1×10^6 cells/ml. The cell suspension was divided into aliquots for different staining panels or controls. Fluorophore-conjugated antibodies specific for CD3 (PE-conjugated, 1:50 dilution; BioLegend), CD4 (APC-conjugated, 1:50 dilution; BioLegend), and anti-mouse CD8a (FITC-conjugated, 1:50 dilution; Invitrogen) antigens were added to the cell suspension. The cells were incubated with the antibodies for 30 minutes at 4 °C, protected from light. After incubation, the cells were washed with staining buffer and centrifuged at 400 × g for 5 minutes at 4 °C. Flow cytometry data were acquired using a Beckman CytoFLEX flow cytometer and analyzed using FlowJo software (version 10.8.1) (Supplementary Fig. 26).

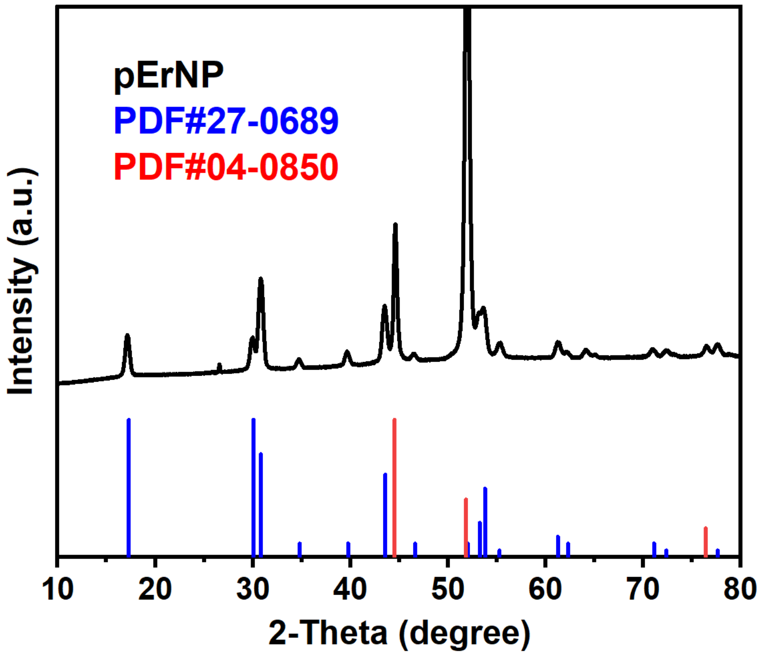

Supplementary Fig. 1 I X-ray diffraction (XRD) patterns of hexagonal β-phase pErNPs ($NaErF_4$/$NaYF_4$ core-shell nanoparticles) nanoparticles. To prepare the pErNP sample for XRD analysis, a 100 mg/ml solution of pErNPs in cyclohexane was used. The solution was then dropped onto a Ni foil to form a thin layer. The XRD patterns of the pErNP sample were measured using a Rigaku Miniflex 600 Benchtop instrument with Cu-Kα radiation. The Joint Committee on Powder Diffraction Standards (JCPDS) card files for $NaErF_4$ (PDF#27-0689) and for Ni (PDF#04-0850) are shown below the spectra for phase identification. a.u., arbitrary units.

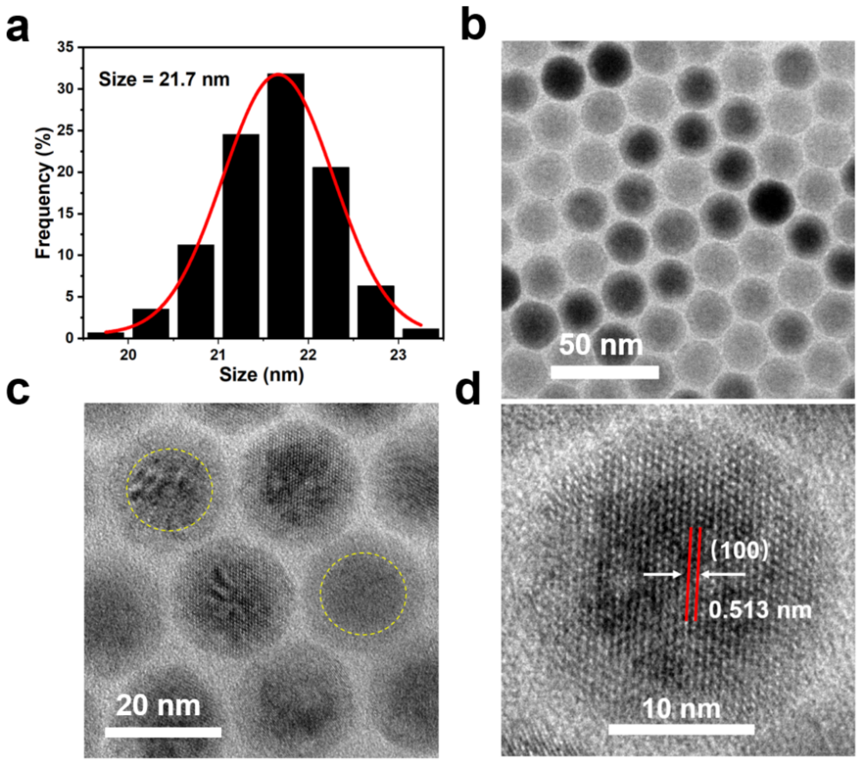

Supplementary Fig. 2 l (a) The histograms of pErNP TEM images show that the size of the nanoparticles is ~ 21.7 nm. (b) Low-resolution TEM images of pErNP. (c) and (d) High-resolution TEM images of pErNP revealing the core/shell structure and clear lattice fringes with a distance of 0.513 nm corresponding to the d-spacing of the (100) lattice planes of the hexagonal structure. The shell thickness is about 3.8 nm. To prepare the pErNP sample for TEM analysis, a 10 mg/ml solution of pErNPs in cyclohexane was dropped onto a 400-mesh copper carbon film. The sample was imaged using a FEI Tecnai G2 F20 X-TWIN Transmission Electron Microscope.

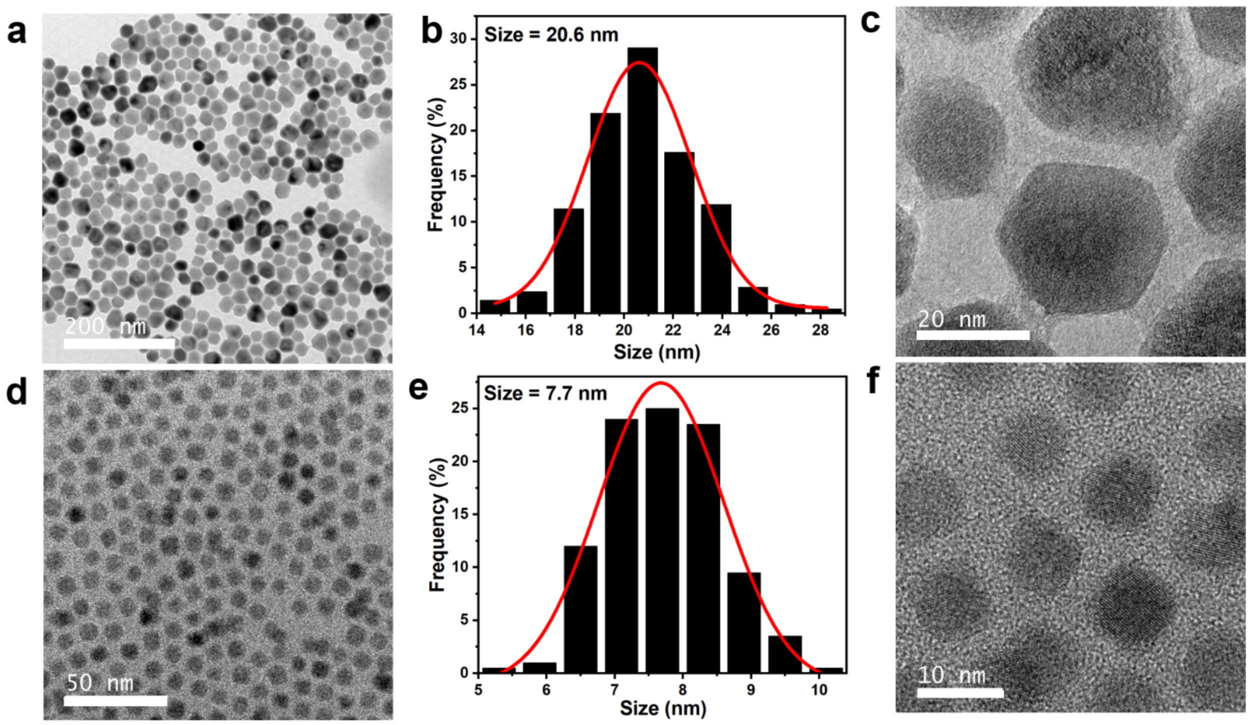

Supplementary Fig. 3 l (a) TEM images of the ErNP[7] and their corresponding size histograms (b). (c) shows the high-resolution TEM images of ErNP. (d) TEM images of the QD and their corresponding size histogram (e). (f) shows the high-resolution TEM images of QD. To prepare the QD sample for TEM analysis, a 2 mg/ml solution of QDs in toluene was dropped onto a 400-mesh copper carbon film. The sample was imaged using a FEI Tecnai G2 F20 X-TWIN Transmission Electron Microscope. ErNP was measured using the same procedure as used for imaging the pErNP sample.

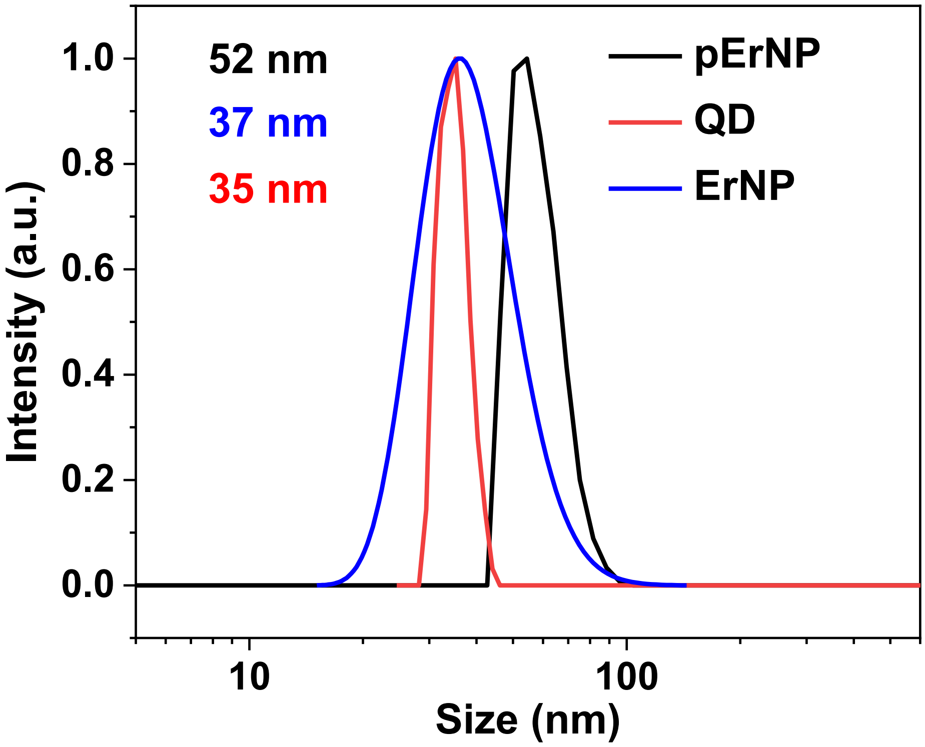

Supplementary Fig. 4 l Dynamic light scattering (DLS) spectra of hydrophilic pErNP-P^3, QD-P^3 and ErNP-P^3 measured in PBS buffer. The average hydrated sizes for these three particles are 52 nm, 35 nm, and 37 nm, respectively. The samples were analyzed by DLS in PBS buffer using a Malvern Zetasizer Nano ZS90.

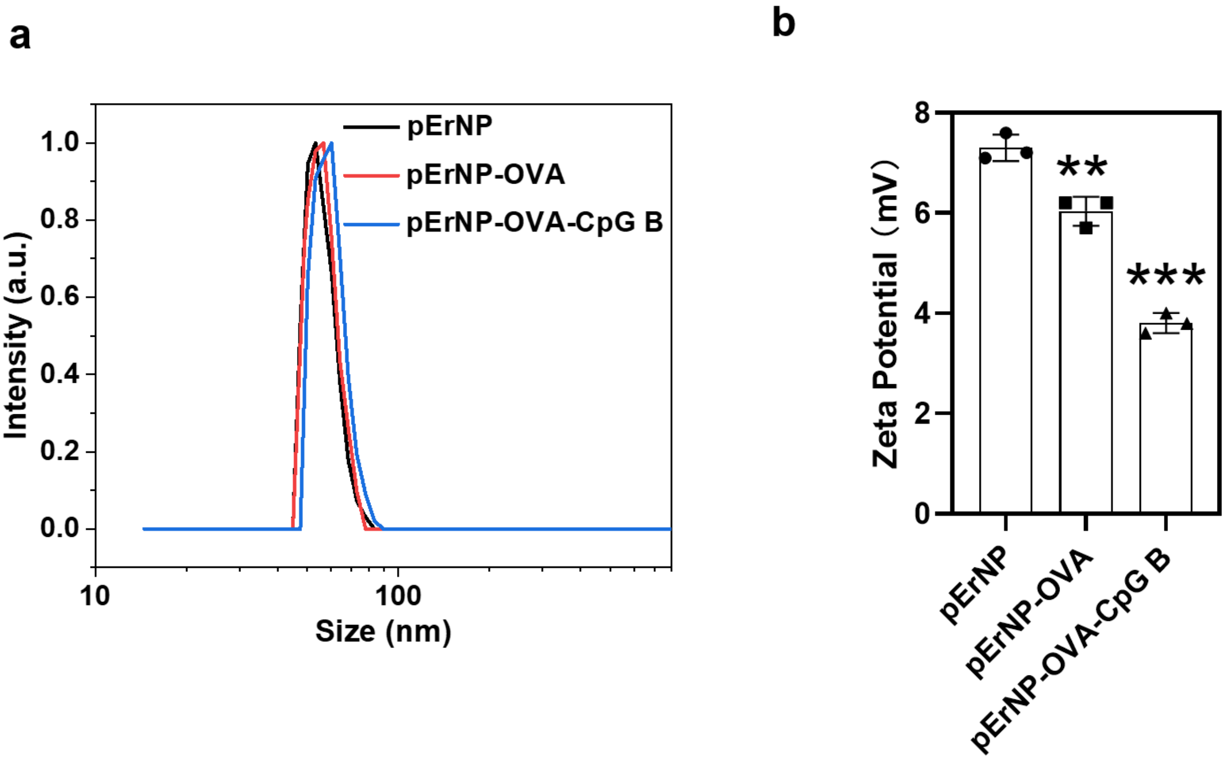

Supplementary Fig. 5 l (a) DLS spectra of hydrophilic P³ coated pErNP, pErNP-OVA and pErNP-OVA-CpG B in PBS buffer. (b) The zeta potentials of the three nanoparticles measured in PBS buffer. The samples were analyzed by DLS and zeta potential measurements in PBS buffer using a Malvern Zetasizer Nano ZS90 (n = 3). pErNP-OVA versus pErNP, p = 0.0050; pErNP-OVA-CpG B versus pErNP-OVA, p = 0.0004. Data are presented as mean ± s.d. and analysed by two-tailed Student's t-test.

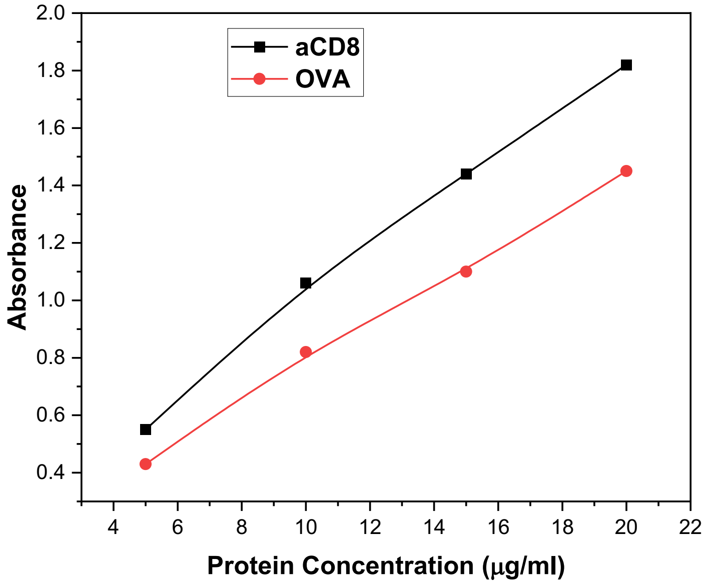

Supplementary Fig. 6 | We used the Pierce BCA protein assay kit to measure the total protein concentration in a sample. The curves here show UV vis absorbance values measured at 562 nm due to Cu(I) ions generated by OVA and aCD8 of various concentrations reacting with Cu(II) ions in BCA assays. These curves are used as calibration curves for analyzing unconjugated OVA or aCD8 for analysis of the conjugation efficiencies of OVA and aCD8 to our probes. (See 'Conjugation efficiencies of various biomolecules on nanoparticles' section). After OVA conjugation and filtering to retain the conjugate, the measured absorbance was 0.42 corresponding to 4.4 μg/ml of unconjugated OVA in the 1 ml filtrate. Thus, the conjugation efficiency was calculated to be approximately 95.6%. Similarly, for the anti-CD8α diabody conjugation, the measured absorbance was 0.35 corresponding to 3.3 μg/ml of unconjugated diabody in the 1 ml filtrate, suggesting a conjugation efficiency of approximately 94.5%.

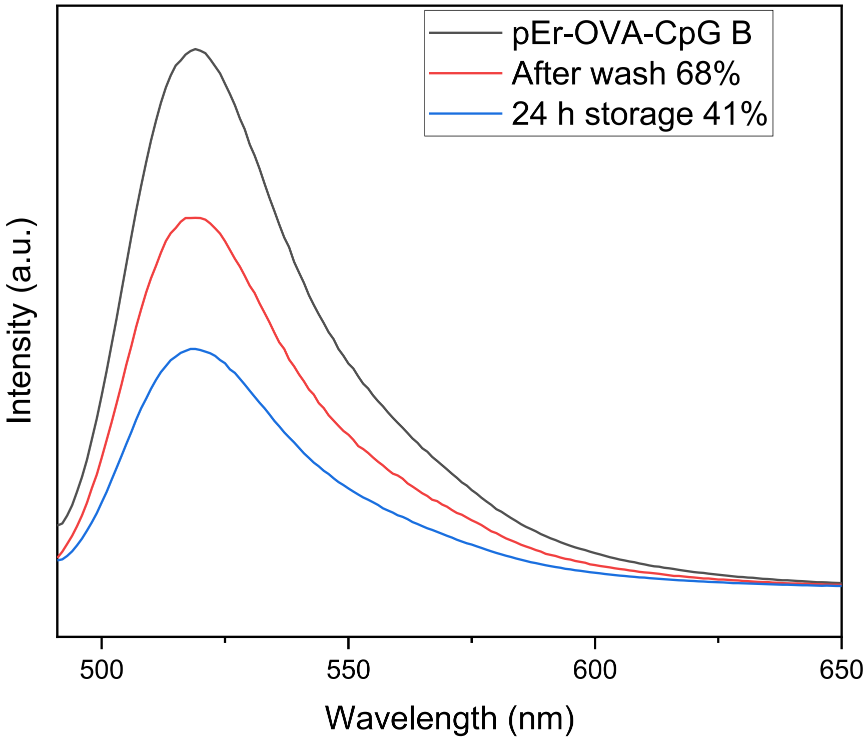

Supplementary Fig. 7 I Emission spectra of pErNP-OVA-CpG B-FITC for analysis of CpG B bound to the complex. The OVA conjugated pErNP (2 mg pErNP, and 100 µg OVA) and FITC labeled CpG B (5 µg) were mixed together and then incubated at room temperature for 30 min. The resulting mixture was washed using a 100kDa centrifugal filter with 1x PBS buffer by centrifuging at 4400 rpm for 10 minutes, four times. We found that 68% of the FITC-CpG B remained bound to pErNP-OVA after the rigorous washing process. After storing the solution for 24 hours, we repeated the washing step and found that 41% of the FITC-CpG B remained bound to pErNP-OVA.

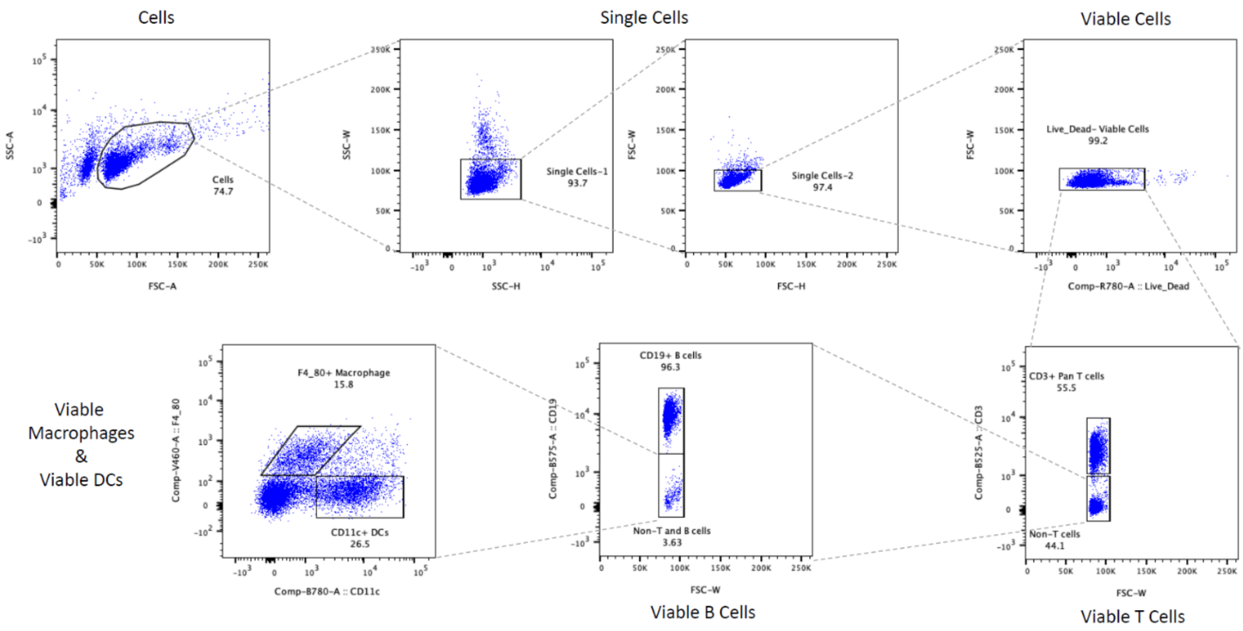

Supplementary Fig. 8 | Cell sorting logic for inguinal lymph node immune cells. 24 hours after vaccination with pErNP-OVA-CpG B, the iLNs were harvested, mechanically disassociated, and processed into a single cell suspension, followed by ACK lysis to remove red blood cells. The cells were then stained with anti-mouse CD3-AF488 (pan T cells), anti-mouse CD19-PE (B cells), anti-mouse CD11c-PE/Cy7 (DCs), anti-mouse F4/80-BV421 (macrophages) for immune phenotyping and Live/Dead® Fixable Near IR dye for viability. Sequential gates were used to identify single cells based on forward and side scatter signals and then viable cells based on Live/Dead staining, followed by the specific immune cell populations, based on expression of the lineage markers. The stained cells were then sorted using BD FACSAria II sorter. The enumeration of cell types after sorting is shown on Supplementary Table 1.

Counts	T cells	B cells	Dendritic cells	Macrophages
iLN M1	2,365,060	1,654,771	16,777	5,171
iLN M2	2,838,292	2,444,778	18,740	7,055
iLN M3	423,3843	3,316,787	36,429	14,860

Supplementary Table 1 I Enumeration of T cells, B cells, dendritic cells, and macrophages purified by cell sorting from iLNs of pErNP-OVA-CpG B and pErNP-P3 treated mice (n = 3, M1-M3 indicate individual mice). 24 hours after vaccination with pErNP-OVA-CpG B, the iLNs were removed and processed into single-cell suspensions. The cells were stained with anti-mouse CD3-AF488 (pan T cells), anti-mouse CD19-PE (B cells), anti-mouse CD11c-PE/Cy7 (DCs), and anti-mouse F4/80-BV421 (macrophages) for immune phenotyping and Live/Dead® Fixable Near IR dye for viability. The stained cells were then sorted using BD FACSAria II instrument (see Supplementary Fig. 8 for sorting logic), with the sorted cell count recorded. The results show the cell count of DCs, B cells, T cells, and macrophages in the iLN of pErNP-OVA-CpG B treated mice.

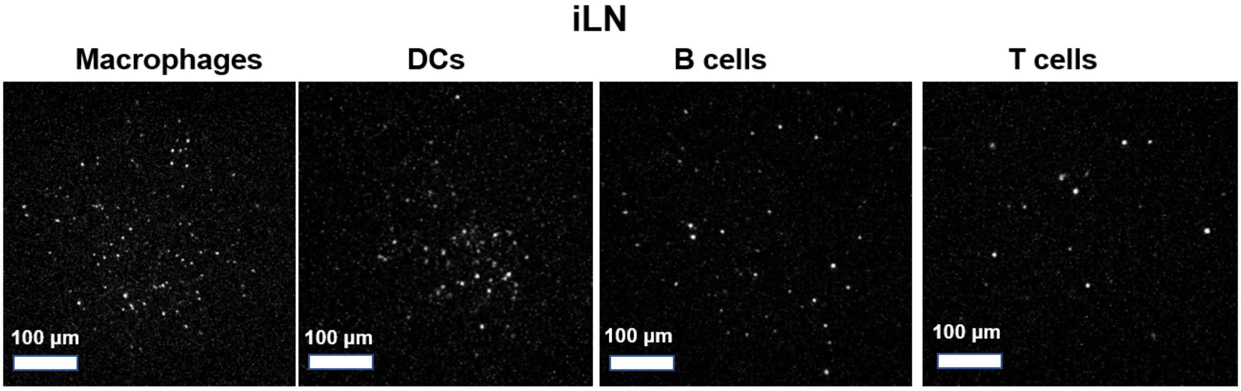

Supplementary Fig. 9 l NIR-IIb imaging of immune cell types, purified from iLNs by fluorescence activated cell sorting. The iLNs were dissected at 24 hours after the injection of pErNP-OVA-CpG B nanovaccine (n = 3 mice). Four types of immune cells, including DCs ($CD11c^{+}$), B cells ($CD19^{+}$), T cells ($CD3^{+}$) and macrophages ($F4/80^{+}$), were purified by cell sorting (see details of the method, above and Supplementary Fig. 8 for gating/sorting logic) and imaged using our imaging system in the NIR-IIb window to check the pErNP signal. Imaging conditions: 975 nm excitation, 1500 -1700 nm detection, exposure times 100 ms, CW mode. Representative images from one of three mice are shown.

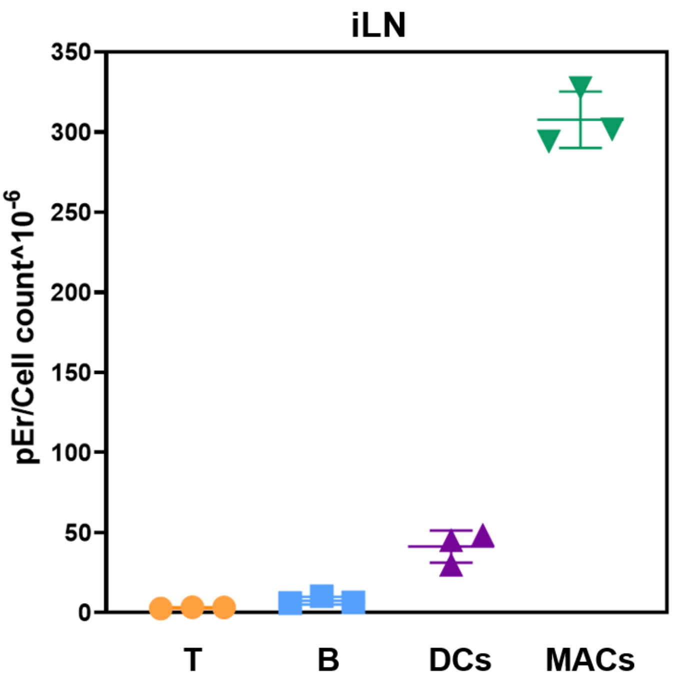

Supplementary Fig. 10 I pEr detected by inductively coupled plasma optical emission spectroscopy (ICP-OES), normalized by cell counts of T cells, B cells, dendritic cells (DC), and macrophages. Cell types were purified from iLNs, extracted from pErNP-OVA-CpG B immunized mice (n=3 mice, values for each mouse are shown). 24 hours after vaccination with pErNP-OVA-CpG B, the iLNs were removed and processed into single cell suspensions. The cells were stained with anti-mouse CD3-AF488 (pan T cells), anti-mouse CD19-PE (B cells), anti-mouse CD11c-PE/Cy7 (DCs), and anti-mouse F4/80-BV421 (macrophages) for immune phenotyping and Live/Dead® Fixable Near IR dye for viability. The stained cells were then sorted using BD FACSAria II sorter (see Supplementary Fig. 8 for sorting logic), with the sorted cell counts recorded. We then measured the pEr content in each cell type using ICP-OES. Briefly, the cells were collected by centrifugation and digested in nitric acid (68%) for 12 hours. The concentration of Er element in each sample was measured using a Thermo Scientific ICAP 6300 Duo View Spectrometer. Ratios of pEr amount to cell count of each immune cell population were calculated. Results show that the DCs had an average pEr content 13.6 times higher than T cells, the macrophages had an average pEr content 101 times higher than T cells, and the B cells had an average pEr content 2.1 times higher than T cells. This suggests that antigen presenting cells efficiently take up pErNP-OVA-CpG B vaccine. (For detailed information, see Supplementary Tables 1 and 2).

Er content (in units of ppb)	T cells	B cells	Dendritic cells	Macrophages
iLN M1	5.97	16.98	0.76	1.52
iLN M2	9.16	14.35	0.56	2.31
iLN M3	14.2	21.14	1.77	4.48

Supplementary Table 2 I The pEr content was measured by ICP-OES in immune cells isolated by cell sorting. We purified immune cells from the iLNs of mice treated with pErNP-OVA-CpG (n = 3, M1-M3 indicate individual mice) 24 hours after injection. Four types of immune cells, B cells (CD19+), T cells (CD3+), dendritic cells (CD11c+) and macrophages (F4/80+), were purified by cell sorting using BD FACSAria II instrument (see above for methods, Supplementary Fig. 8 for sorting logic) and quantified. We then measured the pEr content in each cell type using ICP-OES. Briefly, the cells were collected by centrifugation and digested in nitric acid (68%) for 12 hours. The concentration of Er element in each sample was measured using a Thermo Scientific ICAP 6300 Duo View Spectrometer. ppb, parts per billion.

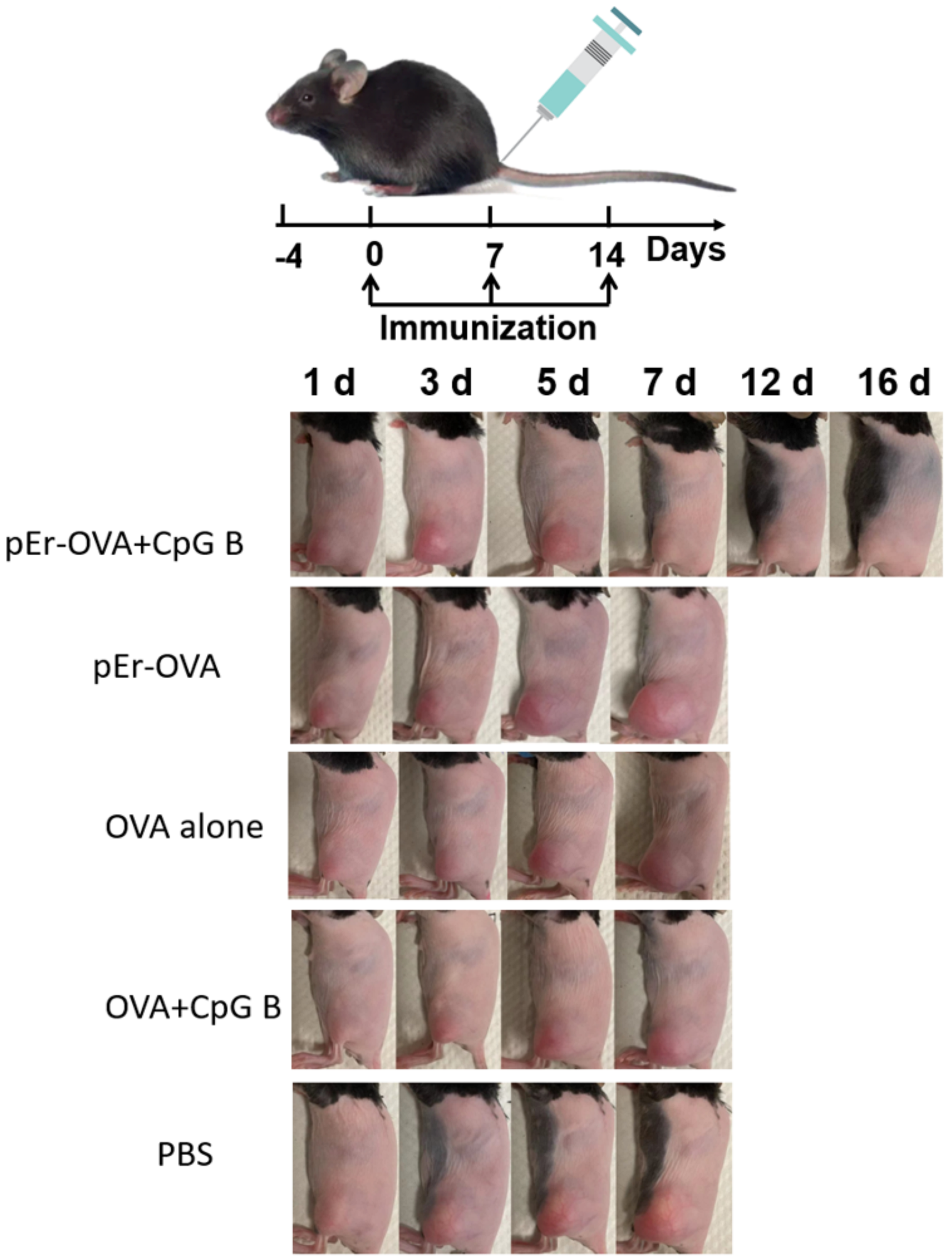

Supplementary Fig. 11 l Photographs of C57BL/6 mice treated with different vaccine formulations: pErNP-OVA-CpG B (n = 17), pErNP-OVA (n = 8), OVA alone (n = 8), OVA+CpG B (n = 5), PBS (n = 12) at different time points post treatment. All mouse groups were injected with the first doses 4 days after E.G7-OVA tumour cell inoculation on the left hindlimb and boosted on days 7 and 14 except for the mice sacrificed due to tumour burden. The mouse group injected with pErNP-OVA-CpG B exhibited the slowest tumour growth, and the tumour volumes started to obviously shrink on ~ day 5 post the first dose. After the booster dose, the tumours continued to shrink over time before disappearing. In contrast, the remaining treatment groups, including PBS, OVA alone, OVA+CPG B and pErNP-OVA mouse groups all showed rapid tumour growth; all of them were sacrificed on day 7 post immunization due to tumour burden.

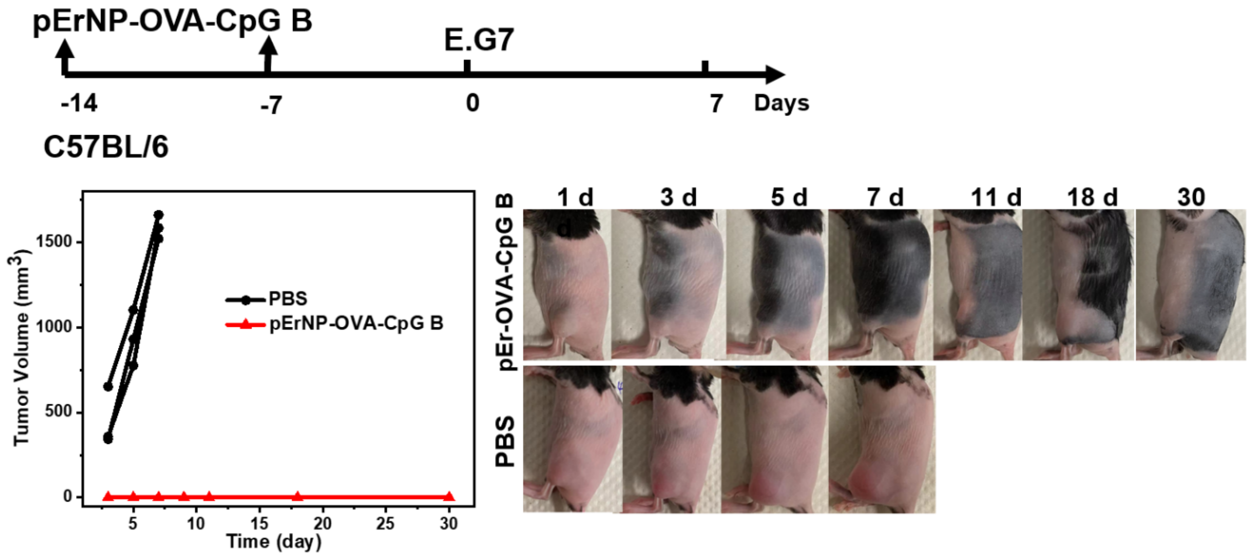

Supplementary Fig. 12 l Tumour challenge for pErNP-OVA-CpG B immunized animal groups (n = 3) and the control animal groups treated with PBS buffer (n=3). C57BL/6 mice were immunized subcutaneously at the base of the tail with 100 µl 1x PBS containing the pErNP-OVA-CpG B (100 µg OVA, 5 µg CpG B and 2 mg pErNP per mouse) or 100 µl 1x PBS. Mice were immunized on days -14 and -7. Seven days later, on day 0, mice were injected subcutaneously with 1×10^{7} E.G7-OVA cells on the left hindlimb. We monitored these mice over 30 days. No detectable solid tumours were grown on mice immunized by two doses of pErNP-OVA-CpG B (photos in the top row). In contrast, the control group treated by two doses of PBS injection showed rapid tumour growth reaching ~ 1500 mm^3 volume (photos in bottom row) within 7 days of cancer cell inoculation.

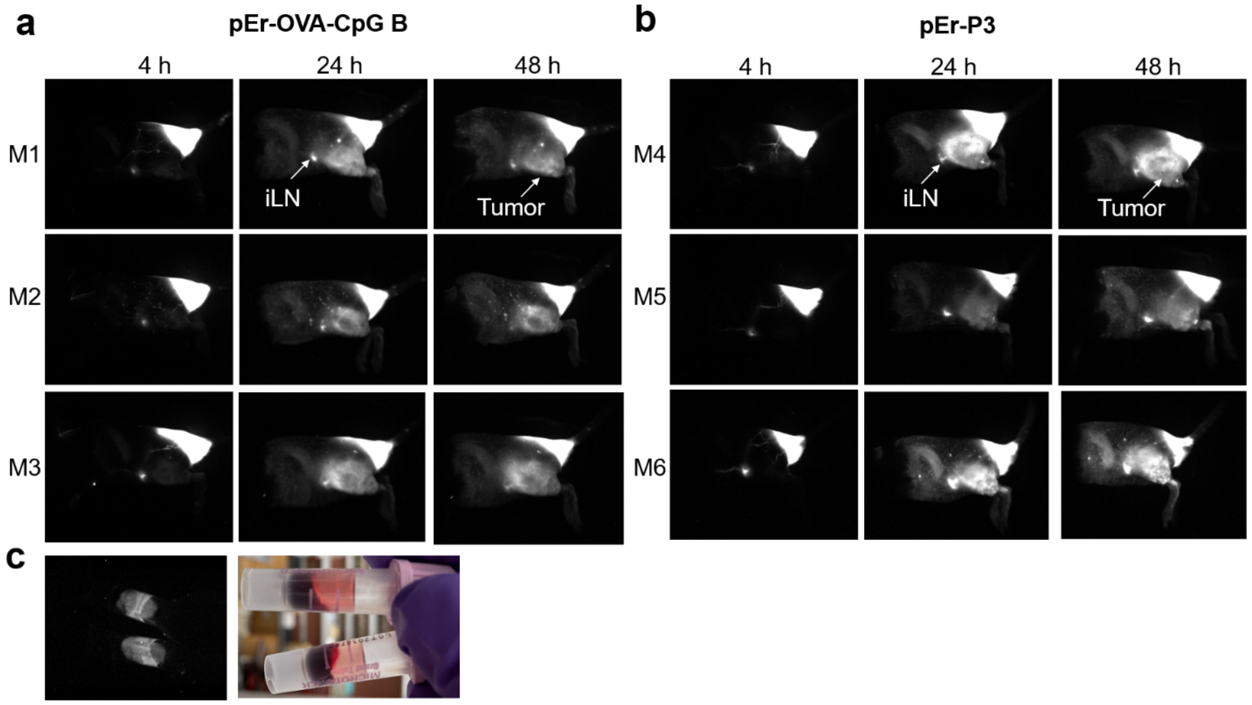

Supplementary Fig. 13 l Wide-field images of mice bearing E.G7 tumours and immunized with pErNP-OVA-CpG B (a) or pErNP-P3 (b) by subcutaneous tail-base injection. (a) Wide-field images of E.G7 tumour-bearing mice (M1-3), immunized with pErNP-OVA-CpG B (975 nm excitation with a power density of ~ 50 mW cm^{-2}, 1500 -1700 nm detection), and imaged at indicated time points post injection. Exposure times are 20 ms for all 4 h time points; for 24 h and 48 h time points, exposure times are 10 ms in CW imaging mode. (b) Wide-field images of E.G7 tumour-bearing mice (M4-6), immunized with pErNP-P3, (975 nm excitation with a power density of ~ 50 mW cm^{-2}, 1500 -1700 nm detection) and imaged at indicated time points post injection. Exposure times are 20 ms for all 4 h time points; for 24 h and 48 h time points, exposure times are 10 ms in CW imaging mode. (c) Left: NIR-IIb image of plasma samples obtained from mice immunized with pErNP-OVA-CpG (upper vial) and pErNP-P3 (lower vial). Right: smartphone image of the two plasma samples in the vials.

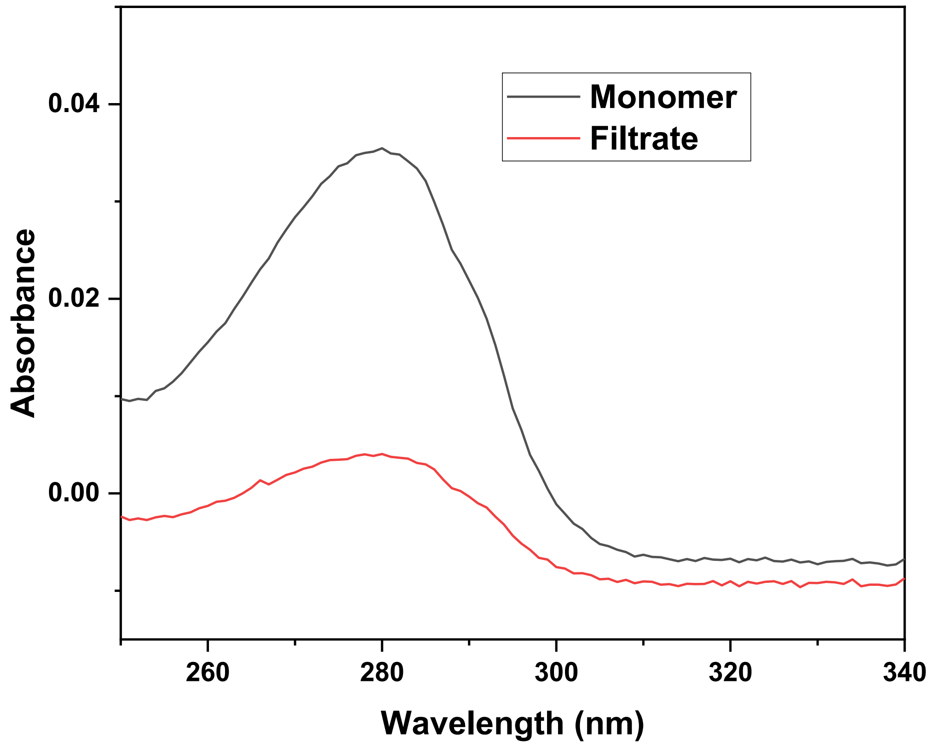

Supplementary Fig. 14 I UV-vis absorption spectroscopy of the SIINFEKL peptide MHC-I H-2K^{b}. The efficiency of tetramer conjugation to QDs was determined by comparing the concentration of monomer in the sample to the total amount of monomer added during the conjugation process. In our experiment, we used 150 μl QD-P3 (in 1x PBS buffer, OD = 1) and 100 μg of biotinylated MHC-I H-2K^{b} chicken OVA$_{257\text{-}264}$ SIINFEKL monomer for conjugation. After filtration and measurement of the absorbance spectrum, we found that the filtrate contained 14 μg of unreacted monomer, resulting in a final conjugation efficiency of approximately 86%.

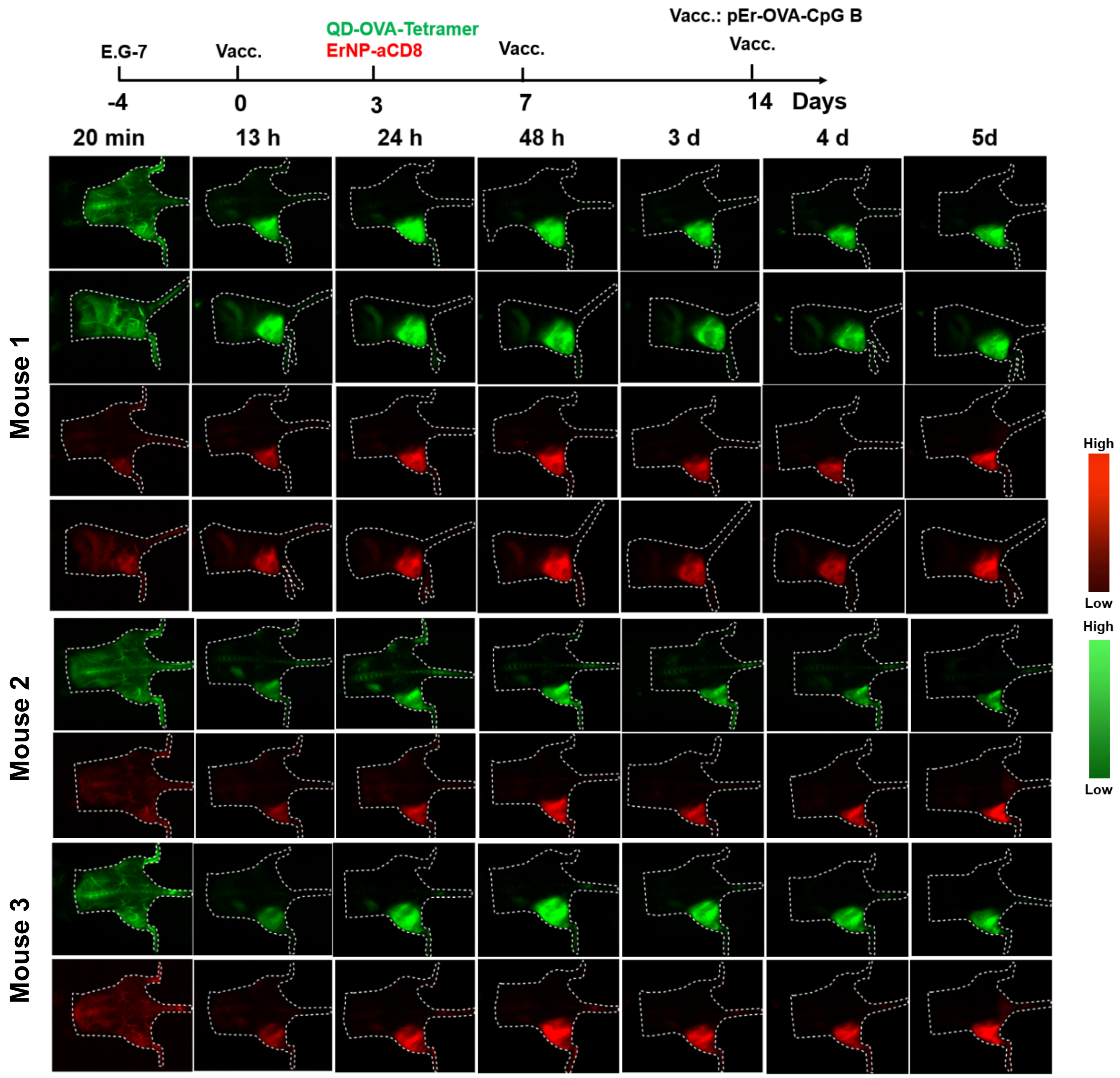

Supplementary Fig. 15 l Three independent data sets of wide-field NIR-IIb molecular images of mice bearing E.G7 tumours immunized with pErNP-OVA-CpG B, recorded at different time points after intravenous injection of ErNP-aCD8 and QD-OVA-Tetramer. (QD-OVA-Tetramer was excited by an 860 nm laser with a power density of ~ 50 mW cm^{-2}, green channel; 1500-1700 nm detection, exposure times 50 ms, CW mode; ErNP-aCD8 was excited by a 940 nm laser with a power density of ~ 50 mW cm^{-2}, red channel; 1500-1700 nm detection, exposure times 20 ms, lifetime mode). Strong signals, corresponding to OVA antigen (green) and specific CD8^{+} (red) CTLs, were observed in the tumours of all three mice immunized with the vaccine.

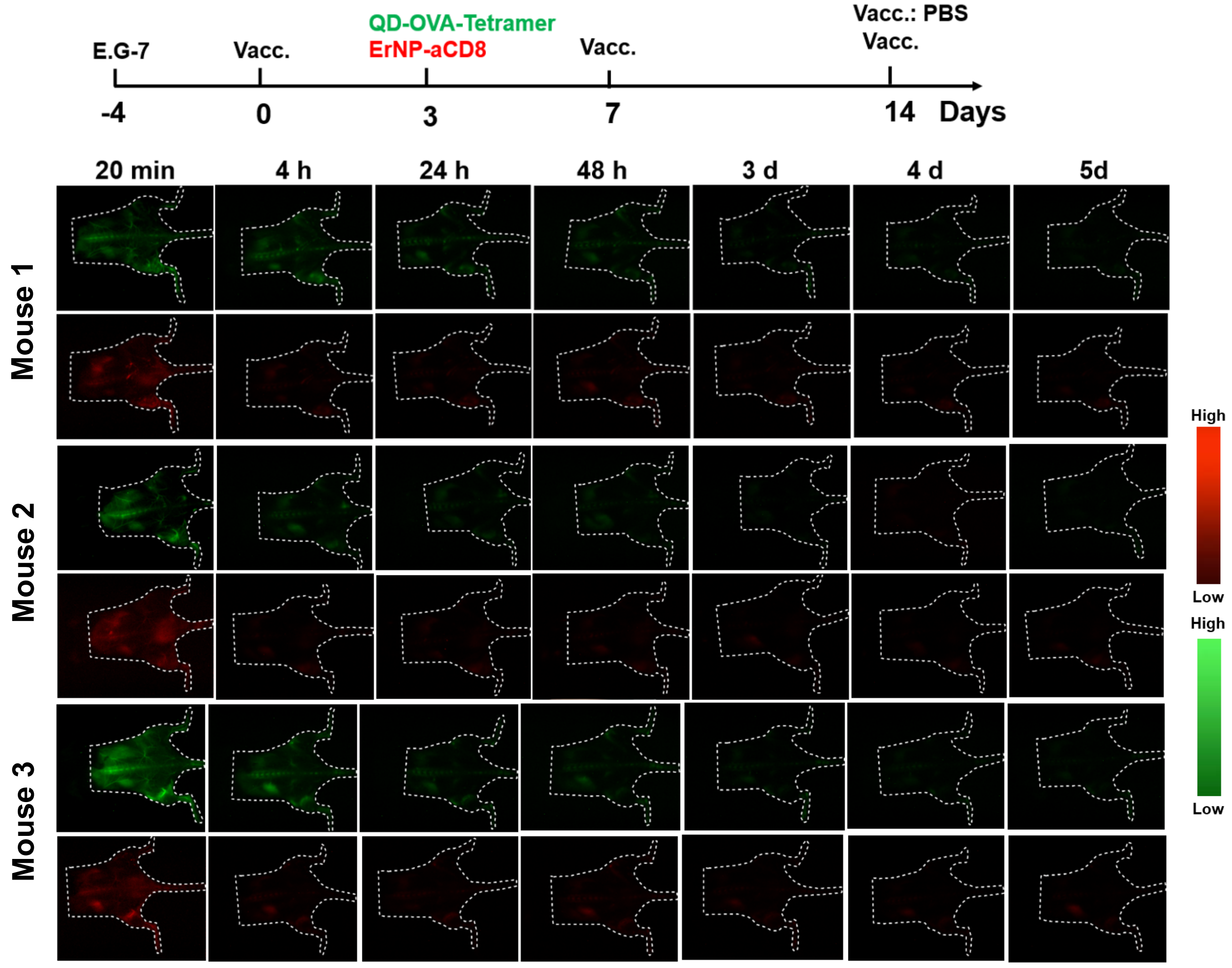

Supplementary Fig. 16 l Three data sets of wide-field NIR-IIb molecular images of mice bearing E.G7 tumours treated with PBS buffer, recorded at different time points after intravenous injection of ErNP-aCD8 and QD-OVA-Tetramer (QD-OVA-Tetramer was excited by an 860 nm laser with a power density of ~ 50 mW cm^{-2}, green channel; 1500-1700 nm detection, exposure times 50 ms, CW mode; ErNP-aCD8 was excited by a 940 nm laser with a power density of ~ 50 mW cm^{-2}, red channel; 1500-1700 nm detection, exposure times 20 ms, lifetime mode). Negligible signals corresponding to OVA antigen (green) or specific CD8+ (red) CTLs were observed in the tumours on all three mice treated by PBS control.

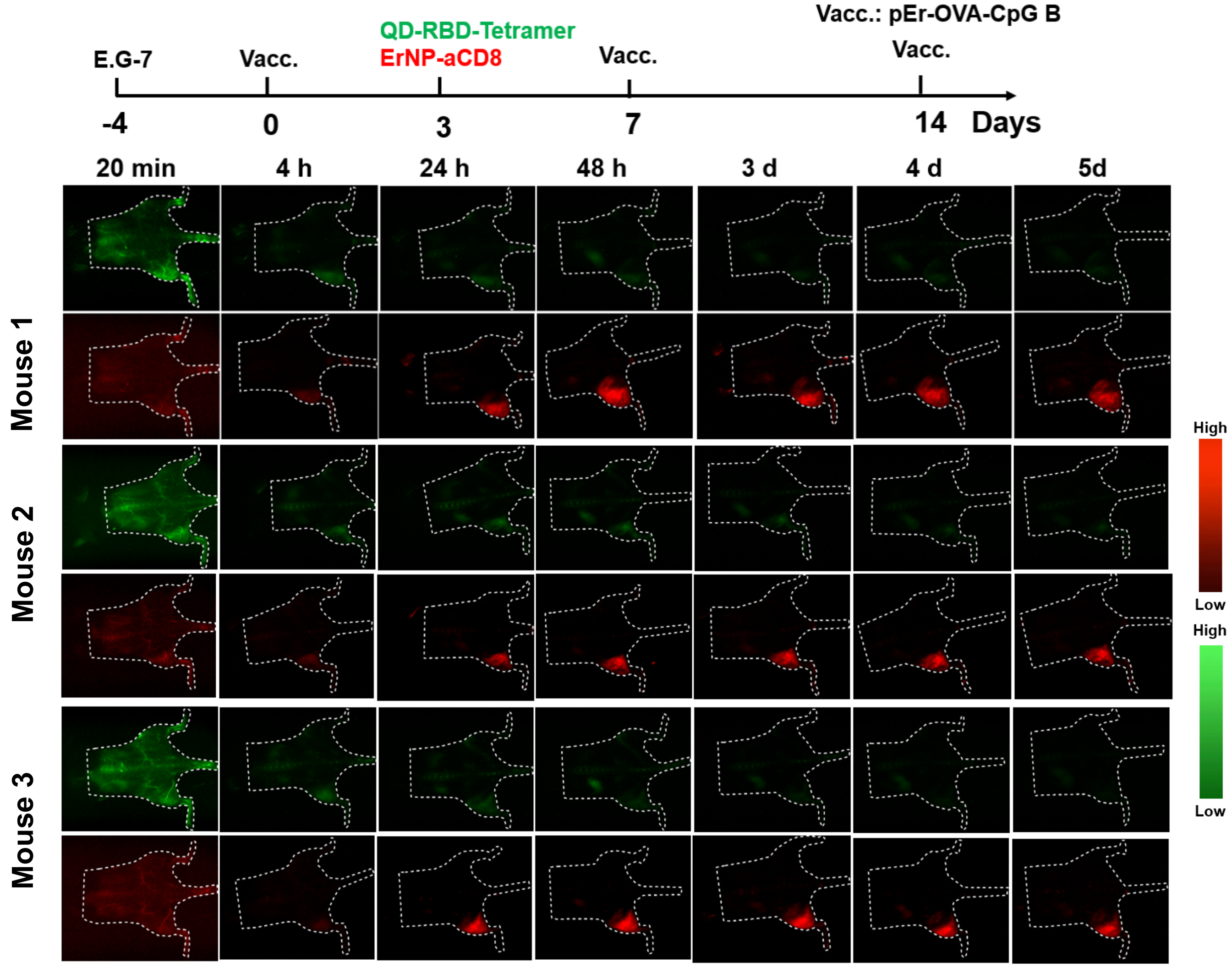

Supplementary Fig. 17 l Three data sets of wide-field NIR-IIb molecular images of mice bearing E.G7 tumours immunized with pErNP-OVA-CpG B, recorded at different time points after intravenous injection of ErNP-aCD8 and QD-RBD-Tetramer (QD-RBD-Tetramer was excited by an 860 nm laser with a power density of ~ 50 mW cm^{-2}, green channel; 1500-1700 nm detection, exposure times 50 ms, CW mode; ErNP-aCD8 was excited by a 940 nm laser with a power density of ~ 50 mW cm^{-2}, red channel; 1500-1700 nm detection, exposure times 20 ms, lifetime mode). Strong CD8 signals were observed in the tumours but RBD-antigen (green) signals were not. The control RBD-Tetramer refers to SARS-CoV-2 $Spike_{539-546}$ VNFNFNGL H-$2K^b$ tetramer.

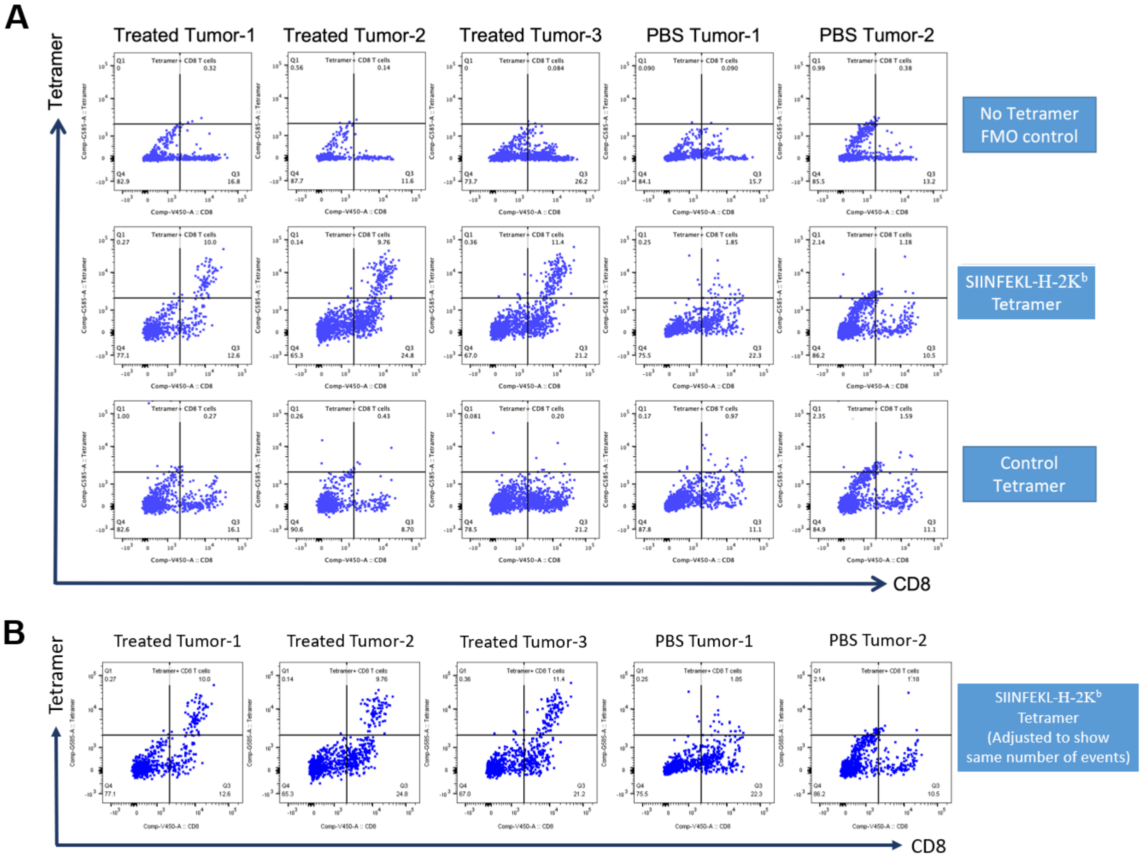

Supplementary Fig. 18 I A. Ex vivo analysis of tumour-infiltrating antigen-specific CD8+ T cells by tetramer staining with protein kinase inhibitor (PKI) dasatinib recovery. Five days after immunization by pErNP-OVA-CpG B nanovaccine (n = 3 mice) or treatment with PBS (n = 2 mice), we quantified OVA-specific SIINFEKL-H-2Kb tetramer+ CD8+ T cells using flow cytometry. SARS-CoV-2 spike RBD-specific VNFNFNGL-H-2Kb tetramer was used as an irrelevant tetramer control, and no tetramer FMO (fluorescence minus one) was also included. Flow cytometry was performed using triplicate samples of tumour-extracted CD45+ cells for each mouse/tetramer condition; a representative plot is shown. Serial gating (Supplementary Fig. 22) identified viable CD3+ T cells in each sample (Supplementary Fig. 23). The percentage of tetramer+ CD8+ T cells among viable CD3+ T cells is shown in the upper right quadrant of each plot. Due to differences in viability in the tumour-extracted cell samples, the number of viable CD3+ T cells is variable between groups. E.G7-OVA tumour cells are CD3+, but CD8neg (Supplementary Fig. 26). Higher frequencies of infiltrating antigen-specific CD8+ T cells are detected in tumours of vaccinated mice.
B. Flow cytometry data from OVA-specific SIINFEKL-H2Kb tetramer and anti-CD8 staining with the same number of events shown in each plot. Higher frequencies of infiltrating antigen-specific CD8+ T cells are detected in tumours of vaccinated mice.

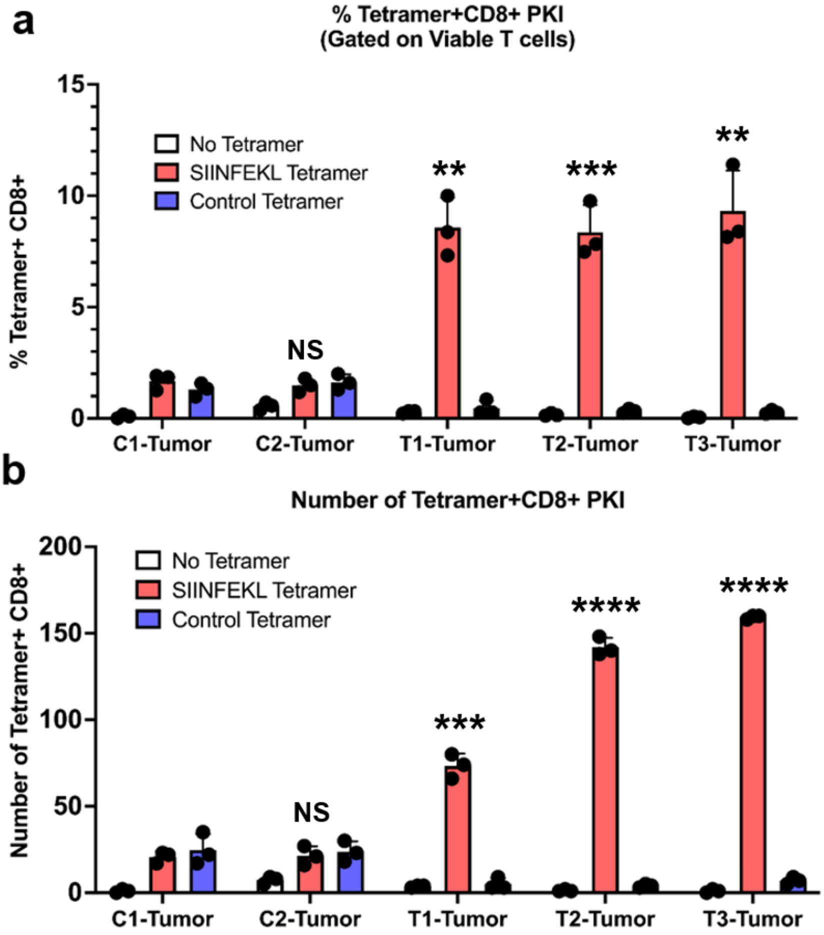

Supplementary Fig. 19 l Frequency (a) and enumeration (b) of tetramer$^+$ CD8$^+$ T cells among viable CD3$^+$ T cells with dasatinib (PKI) recovery. Cells were isolated from tumours 5 days after treatment with pErNP-OVA-CpG B nanovaccine (n = 3, T1-T3 indicate individual treated mice) or PBS buffer (n = 2, C1-C2 indicate individual control mice), then stained for SIINFEKL-H-2K^b tetramer$^+$ CD8$^+$ T cells (red) quantification. 50 nM dasatinib (PKI) was used to optimize tetramer staining[5,6]. No tetramer (white) and SARS-CoV-2 spike RBD-specific VNFNFNGL-H-2K^b tetramer (blue) controls were used for staining specificity. Flow cytometry and analysis were performed using triplicate samples of tumour-extracted cells for each condition; each dot represents a sample. More SIINFEKL-H-2K^b tetramer$^+$ CD8$^+$ T cells were detected in tumours from pErNP-OVA-CpG B nanovaccine treated mice compared to the tumours of PBS controls. For SIINFEKL Tetramer in Supplementary Fig. 19a, C2-Tumour versus C1-Tumour, P = 0.5340; T1-Tumour versus C1-Tumour, P = 0.0010; T2-Tumour versus C1-Tumour, P = 0.0008; T3-Tumour versus C1-Tumour, P = 0.0020. For SIINFEKL Tetramer in Supplementary Fig. 19b, C2-Tumour versus C1-Tumour, P = 0.8651; T1-Tumour versus C1-Tumour, P = 0.0003; T2-Tumour versus C1-Tumour, P < 0.0001; T3-Tumour versus C1-Tumour, P < 0.0001. All data are from three independent experiments and are presented as means ± s.d. Two-sided Student's t-tests were used for the comparisons.

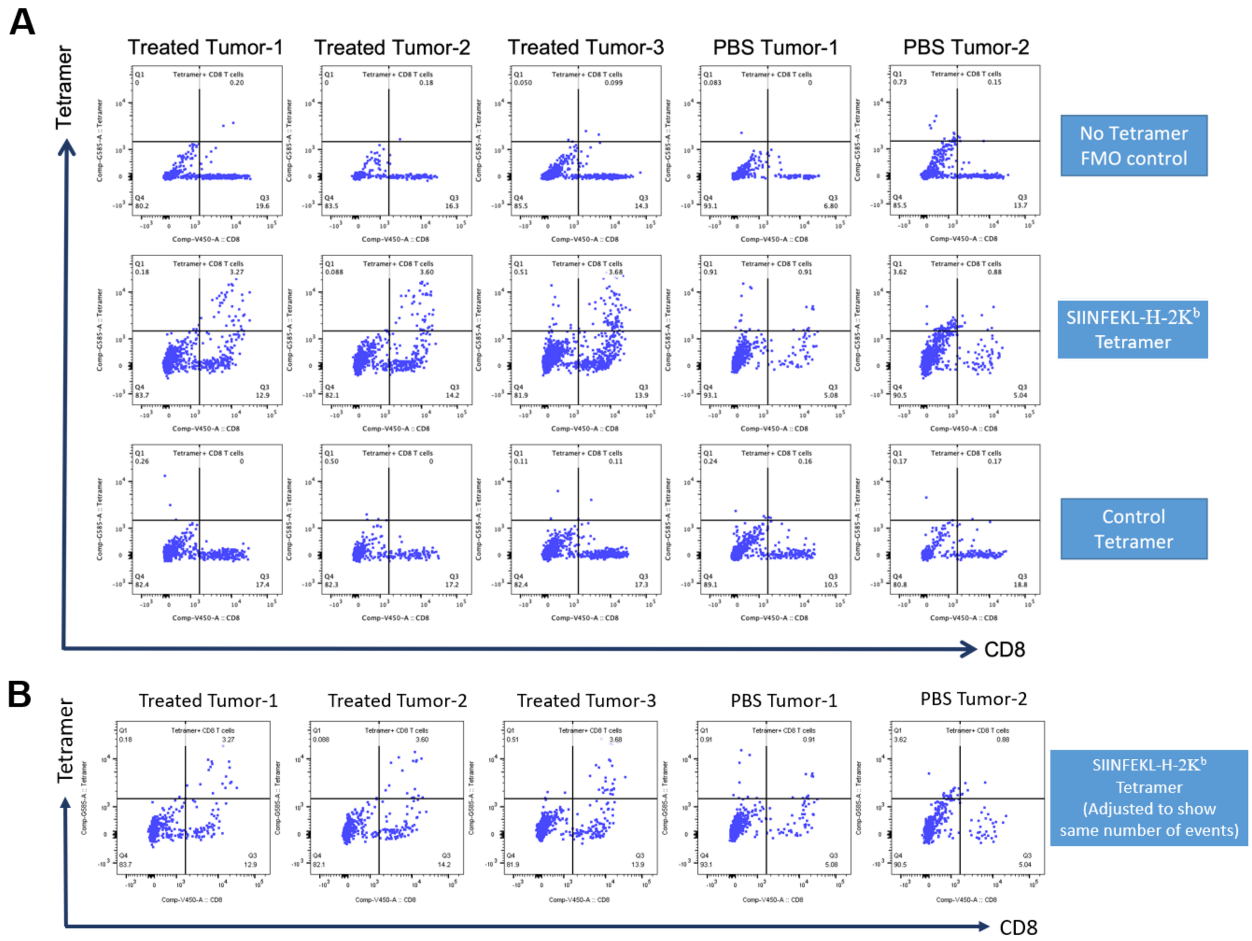

Supplementary Fig. 20 I Ex vivo analysis of tumour-infiltrating antigen-specific $CD8^+$ T cells by tetramer staining without PKI dasatinib recovery. Five days after immunization by pErNP-OVA-CpG B nanovaccine (n = 3) or treatment with PBS (n = 2), the OVA-specific SIINFEKL-H2Kb $tetramer^+$ $CD8^+$ T cells were quantified using flow cytometry. SARS-CoV-2 spike RBD-specific VNFNFNGL- $H\text{-}2K^b$ tetramer was used as an irrelevant tetramer control and no tetramer FMO was also included. Flow cytometry was performed using triplicate samples of tumour-extracted CD45+ cells for each mouse/tetramer condition; a representative plot is shown. Serial gating (Supplementary Fig. 24) identified viable $CD3^+$ T cells in each sample (Supplementary Fig. 25). The percentage of $tetramer^+$ $CD8^+$ T cells among viable $CD3^+$ T cells is indicated in the upper right quadrant of each representative dot plot. Due to differences in viability in the tumour-extracted cell samples, the number of viable CD3+ T cells is variable between groups. E.G7-OVA tumour cells are $CD3^+$, but $CD8^{neg}$ (Supplementary Fig. 26). Higher frequencies of infiltrating antigen-specific $CD8^+$ T cells are detected in tumours of vaccinated mice.

B. Flow cytometry data from OVA-specific SIINFEKL-$H2K^b$ tetramer and anti-CD8 staining with the same number of events shown in each plot. Higher frequencies of infiltrating antigen-specific $CD8^+$ T cells are detected in tumours of vaccinated mice.

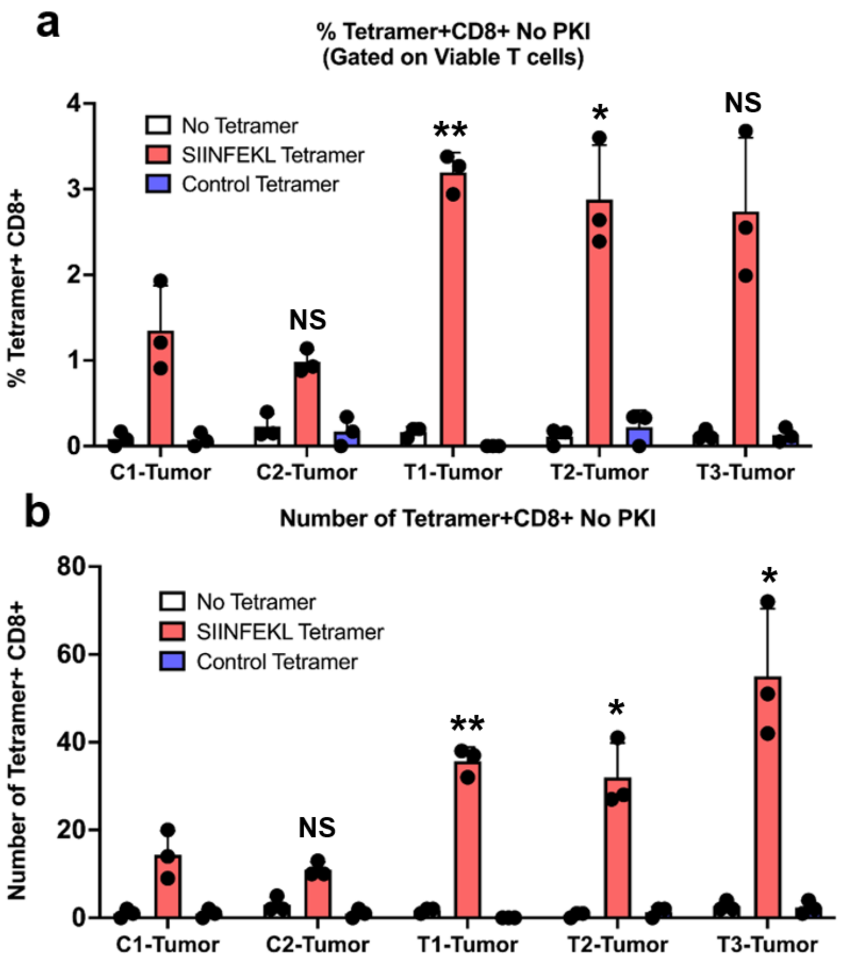

Supplementary Fig. 21 I Frequency (a) and enumeration (b) of tetramer+ CD8+ among viable T cells, without PKI dasatinib recovery[5,6]. Cells were isolated from tumours treated with pErNP-OVA-CpG B nanovaccine (n = 3, T1-T3 indicate individual treated mice) or PBS buffer (n = 2, C1-C2 indicate individual control mice) on day 5, then stained for SIINFEKL-H-2K^b tetramer+ CD8+ T cells (red) quantification. No tetramer (white) and SARS-CoV-2 spike RBD-specific VNFNFNGL-H-2K^b tetramer (blue) controls were used for staining specificity. Flow cytometry and analysis were performed using triplicate samples of tumour-extracted cells for each condition; each dot represents a sample. More SIINFEKL-H-2K^b tetramer+ CD8+ T cells were detected in tumours from pErNP-OVA-CpG B nanovaccine-treated mice compared to the tumours of PBS controls. For SIINFEKL Tetramer in Supplementary Fig. 21a, C2-Tumour versus C1-Tumour, $P = 0.3064$; T1-Tumour versus C1-Tumour, $P = 0.0050$; T2-Tumour versus C1-Tumour, $P = 0.0329$; T3-Tumour versus C1-Tumour, $P = 0.0753$. For SIINFEKL Tetramer in Supplementary Fig. 21b, C2-Tumour versus C1-Tumour, $P = 0.3739$; T1-Tumour versus C1-Tumour, $P = 0.0044$; T2-Tumour versus C1-Tumour, $P < 0.0328$; T3-Tumour versus C1-Tumour, $P < 0.0126$. All data are from three independent experiments and are presented as means ± s.d. Two-sided Student's t-tests were used for the comparisons.

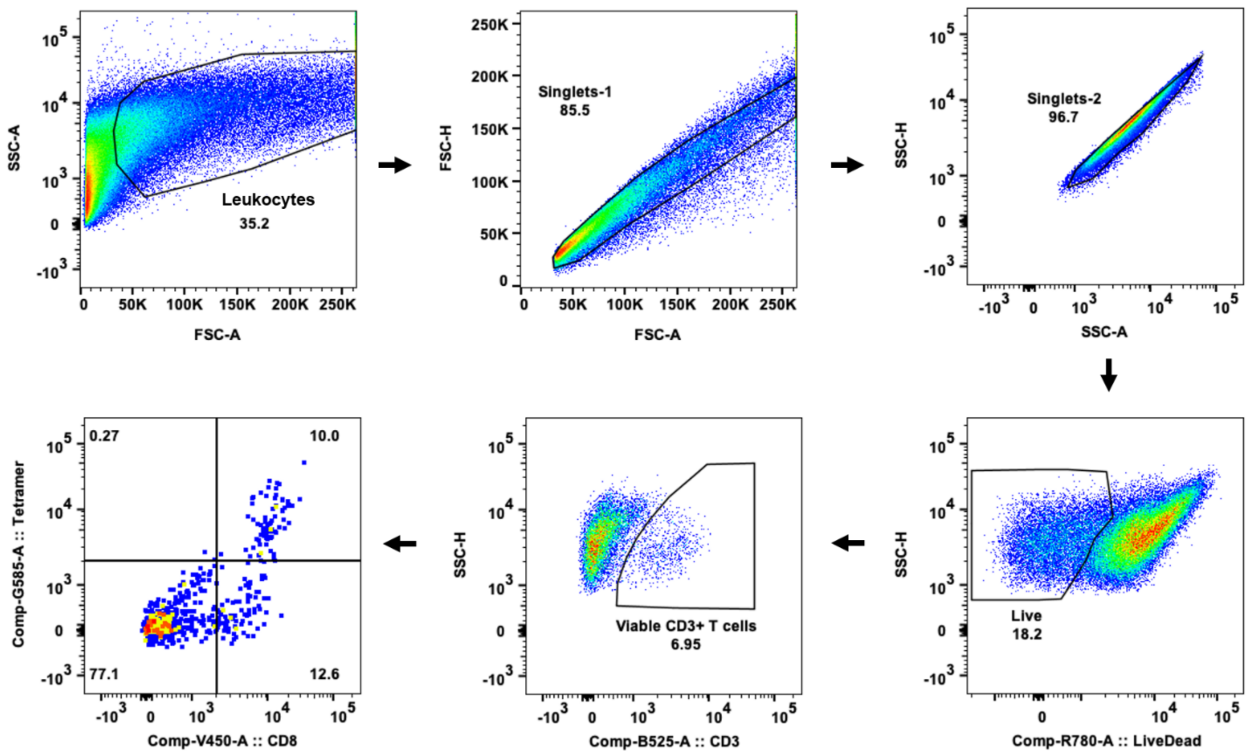

Supplementary Fig. 22 l Flow cytometry gating strategy for tetramer^{+}CD8^{+} cells with PKI dasatinib recovery[5,6]. Sequential gating was applied to stained CD45+ tumour-extracted cell suspensions as follows. First, a polygon gate was applied to FSC-area vs SSC-area to identify the leukocyte population and exclude debris. This size gating included the known size of lymphocytes from the mice. 2×10^5 total leukocyte events were acquired. Then gating based on FSC-area vs FCS-height followed by gating based on SSC-area vs SSC-height was used to identify single cells and exclude cell doublets and aggregates. Live/Dead IR negative cells identified viable cells and viable T cells were identified using a CD3+ polygon gate. E.G7-OVA tumour cells are CD3^{+} CD8^{neg} cells (Supplementary Fig. 26). Infiltrating antigen-specific, CD8^{+} cells were identified as the tetramer^{+}CD8^{+} population within the viable CD3^{+} T cells.

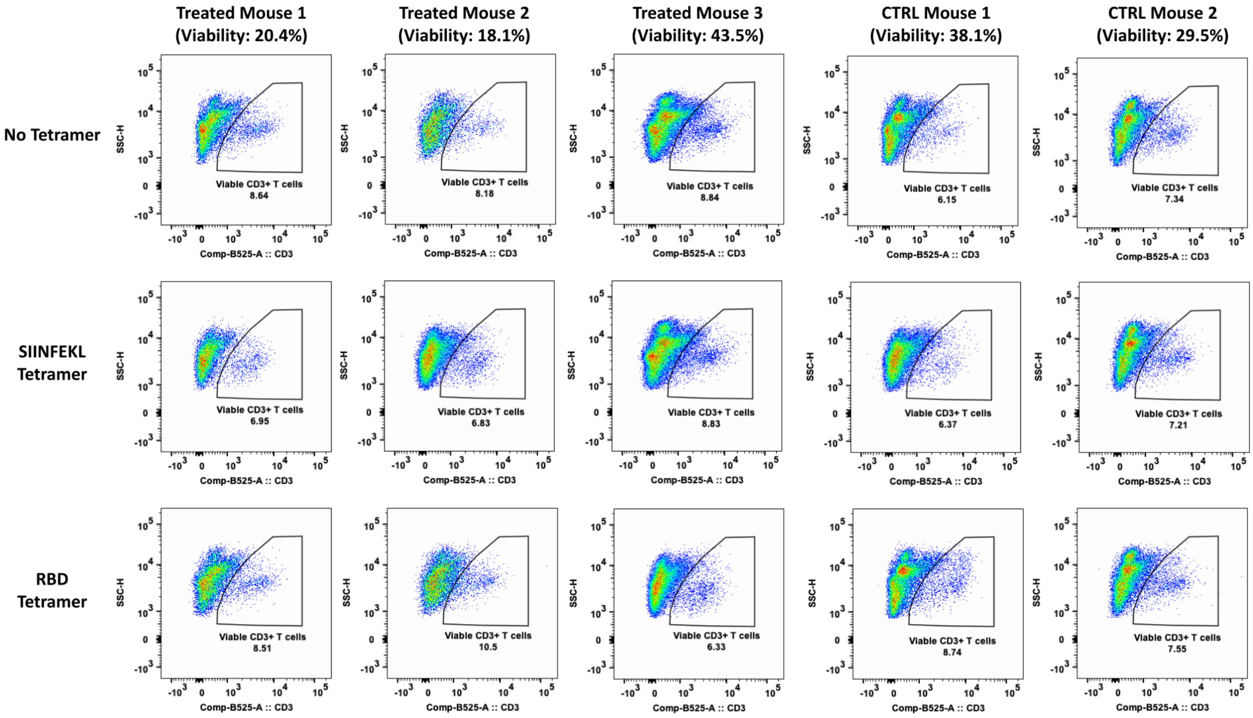

Supplementary Fig. 23 l Frequency of CD3+ viable cells in cells with PKI dasatinib recovery. CD45+ cells were isolated from tumours treated with pErNP-OVA-CpG B nanovaccine (n = 3, T1-T3 indicate individual vaccinated mice) or PBS buffer (n = 2, C1-C2 indicate individual control mice) on day 5. Staining with SIINFEKL or RBD tetramers or no tetramer (as indicated by row) preceded staining with anti-CD3 and anti-CD8 antibodies. Cells were sequentially gated for analysis following the strategy shown in Supplementary Fig. 22. Representative dot plots from triplicate samples show CD3+ cells among viable single cells. Due to differences in total cell viability in the tumour-extracted cell samples, the number of viable CD3+ T cells is variable between samples.

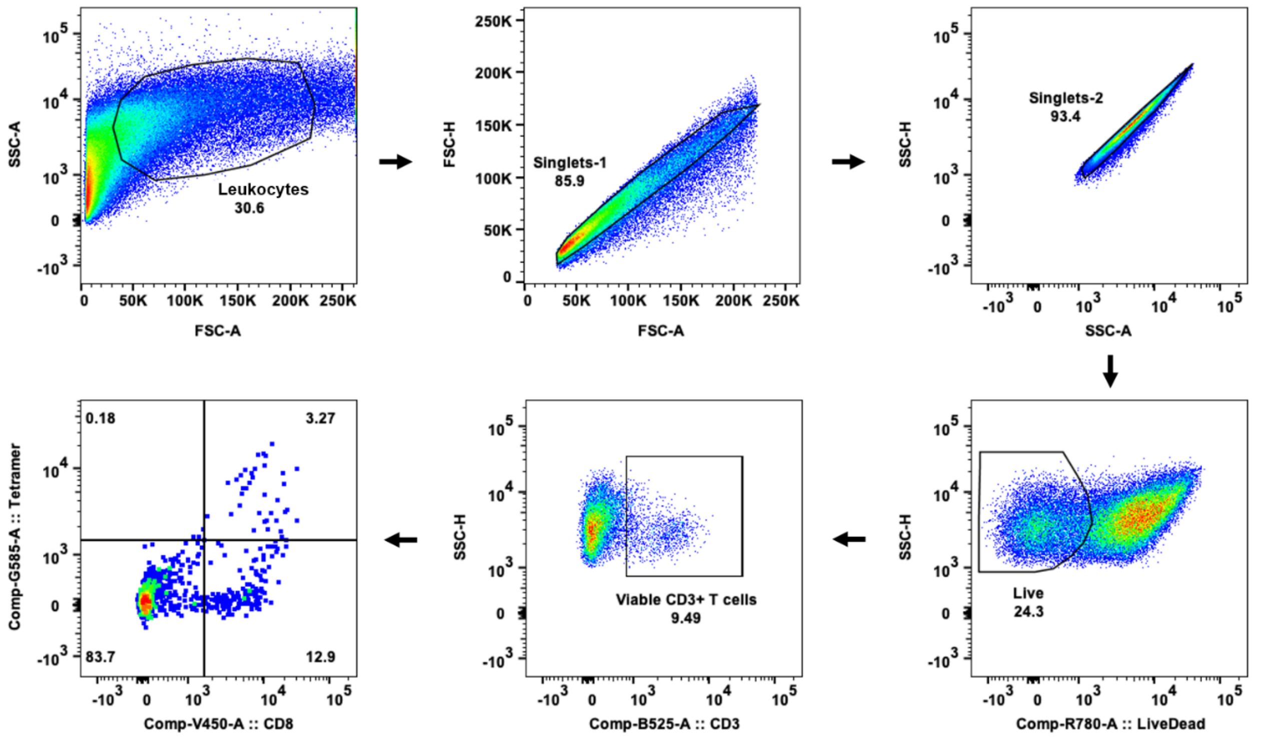

Supplementary Fig. 24 | Flow cytometry gating strategy for tetramer$^+$CD8$^+$ cells without PKI dasatinib recovery. Sequential gating strategies were applied to stained CD45+ tumour-extracted cell suspensions as follows. First, a polygon gate was applied to FSC-area vs SSC-area to identify the leukocyte population and exclude debris. This size gating included the known size of lymphocytes from the mice. 2×10^5 total leukocyte events were acquired. Then gating based on FSC-area vs FCS-height followed by gating based on SSC-area vs SSC-height was used to identify single cells and exclude cell doublets and aggregation. Live/Dead IR negative cells identified viable cells and. viable T cells were identified using a CD3$^+$polygon gate. E.G7-OVA tumour cells are CD3$^+$ CD8^{neg} cells (Supplementary Fig. 26). Infiltrating antigen-specific, CD8$^+$ cells were identified as the tetramer$^+$CD8$^+$ population within the viable CD3$^+$ T cell population.

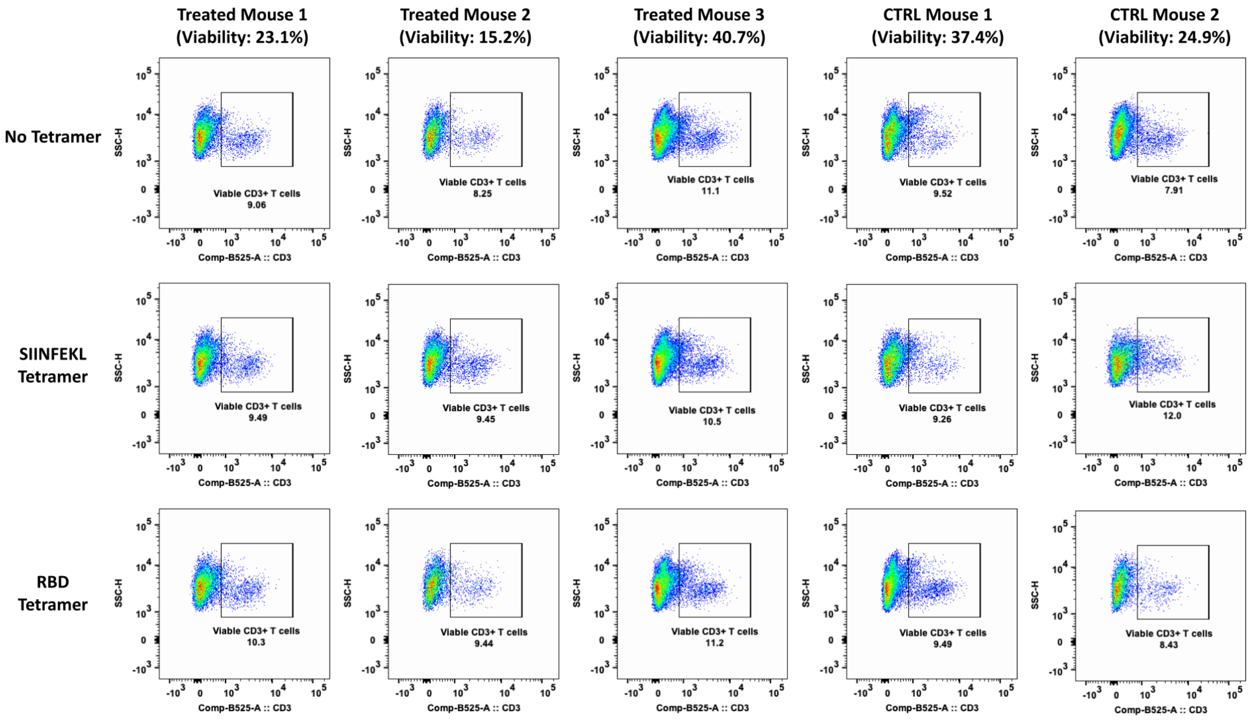

Supplementary Fig. 25 l Frequency of CD3^{+} viable cells in cells without PKI dasatinib recovery. Cells were isolated from tumours treated with pErNP-OVA-CpG B nanovaccine (n = 3, T1-T3 indicate individual vaccinated mice) or PBS buffer (n = 2, C1-C2 indicate individual control mice) on day 5. Staining with SIINFEKL or RBD tetramers or no tetramer (as indicated by row) preceded staining with anti-CD3 and anti-CD8 antibodies. Cells were sequentially gated for analysis following the strategy shown in Supplementary Fig. 24. Representative dot plots from triplicate samples show CD3^{+} cells gated on viable single cells. Due to differences in total cell viability in the tumour-extracted cell samples, the number of viable CD3^{+} T cells is variable between samples.

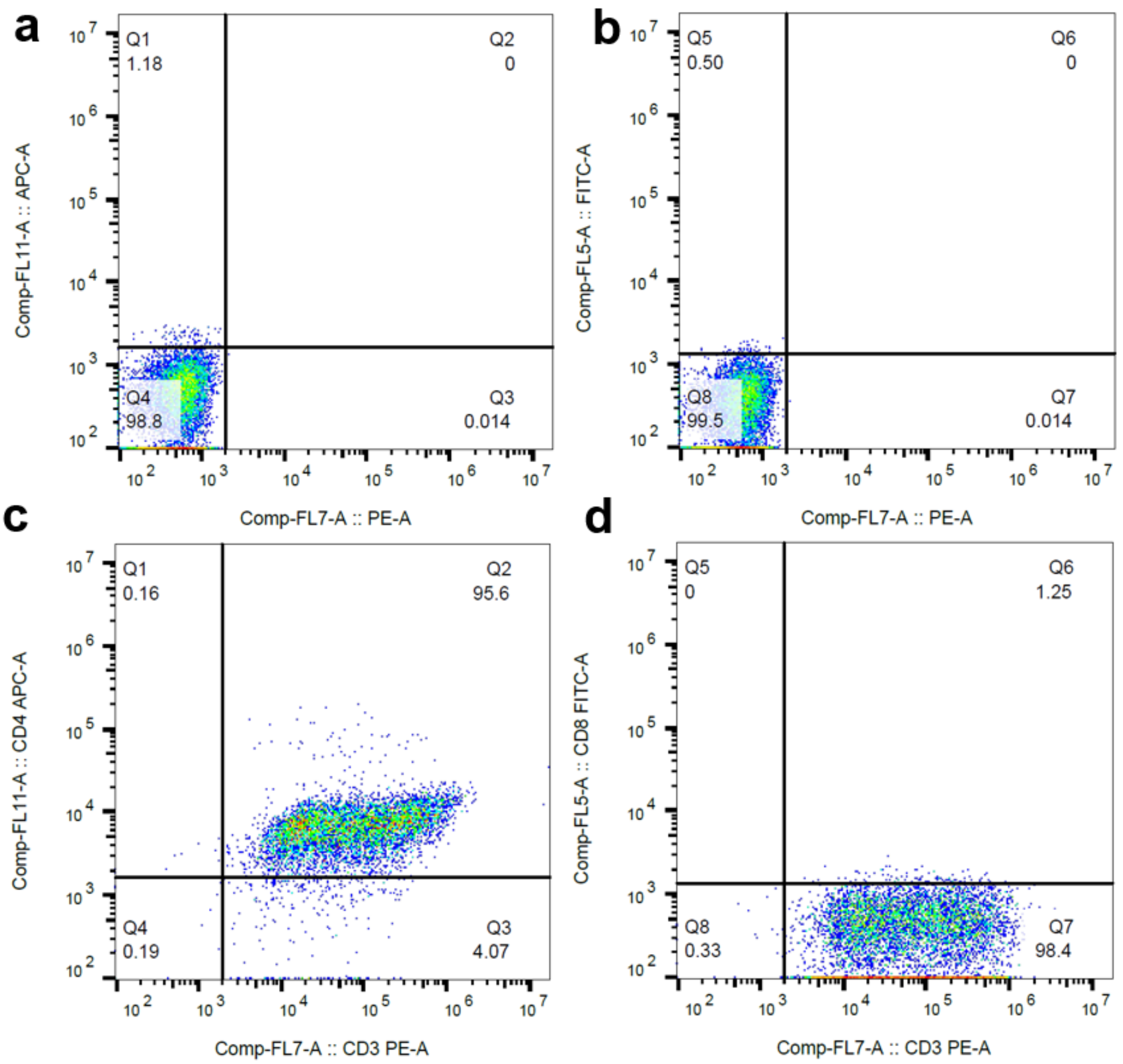

Supplementary Fig. 26 l Flow cytometry analysis of E.G7 tumour cells for CD3, CD4, and CD8 expression. (a) and (b) without any antibodies staining as control groups. (c) CD3 and CD4 expression. (d) CD3 and CD8 expression. E.G7 cells were stained with anti-CD3, anti-CD4, and anti-CD8 antibodies and analyzed by flow cytometry. The results show that E.G7 cells are negative for CD8 and positive for CD3 and CD4 expression.

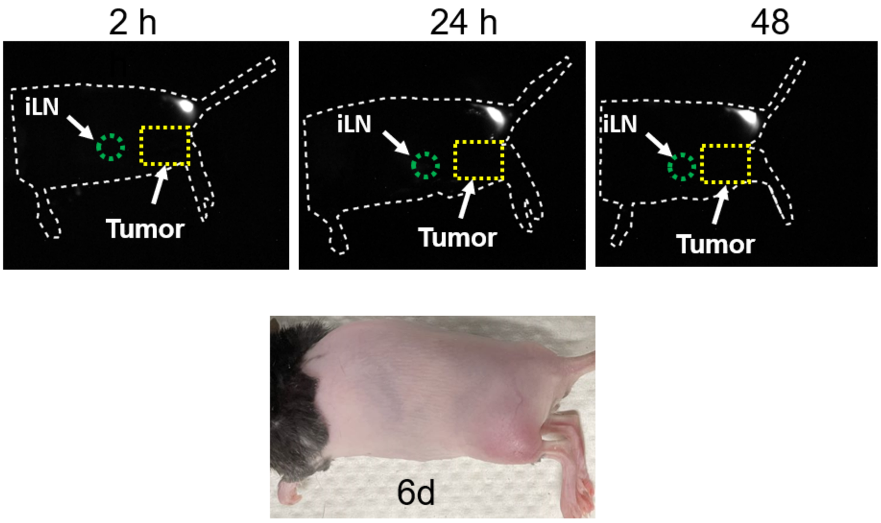

Supplementary Fig. 27 l Wide-field images of mice bearing E.G7 tumours immunized with pErNP-DSPE-OVA-CpG B. pEr-DSPE-OVA-CpG B nanoparticles were prepared using the same method as pEr-P3-OVA-CpG B, except that DSPE-NH_2 was used for surface modification instead of P3. Mice were immunized subcutaneously with pEr-DSPE-OVA-CpG B nanoparticles, and tumour growth was monitored. DSPE PEG (80 mg, 5 kDa) dissolved in 5 ml chloroform was mixed with pErNPs/ErNPs (32 mg) dispersed in cyclohexane. After stirring the solution for 1 h, the organic solvent was evaporated overnight. Then, 6 ml water was added, and the mixture was sonicated to obtain a well dispersed solution of pErNP-DSPE.

References

1. Wang, F. *et al.* In vivo non-invasive confocal fluorescence imaging beyond 1,700 nm using superconducting nanowire single-photon detectors. *Nat Nanotechnol* **17**, 653–660 (2022).
2. Adilakshmi, T. & Laine, R. O. Ribosomal Protein S25 mRNA Partners with MTF-1 and La to Provide a p53-mediated Mechanism for Survival or Death. *Journal of Biological Chemistry* **277**, 4147–4151 (2002).
3. Fischer, T., Elenko, E., McCaffery, J. M., DeVries, L. & Farquhar, M. G. Clathrin-coated vesicles bearing GAIP possess GTPase-activating protein activity *in vitro*. *Proceedings of the National Academy of Sciences* **96**, 6722–6727 (1999).
4. Olson, B. J. S. C. & Markwell, J. Assays for Determination of Protein Concentration. *Curr Protoc Pharmacol* **38**, (2007).
5. Lissina, A. *et al.* Protein kinase inhibitors substantially improve the physical detection of T-cells with peptide-MHC tetramers. *J Immunol Methods* **340**, 11–24 (2009).
6. Dolton, G. *et al.* More tricks with tetramers: A practical guide to staining T cells with peptide-MHC multimers. *Immunology* vol. **146**, 11–22 (2015).
7. Zhong, Y. *et al.* In vivo molecular imaging for immunotherapy using ultra-bright near-infrared-IIb rare-earth nanoparticles. *Nat Biotechnol* **37**, 1322–1331 (2019).
