# Supplementary material for: Shortwave-infrared-light-emitting probes for the in vivo tracking of cancer vaccines and the elicited immune responses
Source: Nat Biomed Eng. 2023 Aug 24;8(6):726–39. doi: 10.1038/s41551-023-01083-5 (PMC11250370; doi:10.1038/s41551-023-01083-5)
Supplement: Supplementary file 2 — Reporting Summary [file 41551_2023_1083_MOESM2_ESM.pdf]

nature portfolio

Corresponding author(s): Hongjie Dai
Last updated by author(s): Jul 22, 2023

Reporting Summary

Nature Portfolio wishes to improve the reproducibility of the work that we publish. This form provides structure for consistency and transparency in reporting. For further information on Nature Portfolio policies, see our Editorial Policies and the Editorial Policy Checklist.

Statistics

For all statistical analyses, confirm that the following items are present in the figure legend, table legend, main text, or Methods section.

n/a	Confirmed	
☐	☒	The exact sample size (*n*) for each experimental group/condition, given as a discrete number and unit of measurement
☐	☒	A statement on whether measurements were taken from distinct samples or whether the same sample was measured repeatedly
☐	☒	The statistical test(s) used AND whether they are one- or two-sided<br>*Only common tests should be described solely by name; describe more complex techniques in the Methods section.*
☒	☐	A description of all covariates tested
☐	☒	A description of any assumptions or corrections, such as tests of normality and adjustment for multiple comparisons
☐	☒	A full description of the statistical parameters including central tendency (e.g. means) or other basic estimates (e.g. regression coefficient) AND variation (e.g. standard deviation) or associated estimates of uncertainty (e.g. confidence intervals)
☐	☒	For null hypothesis testing, the test statistic (e.g. *F*, *t*, *r*) with confidence intervals, effect sizes, degrees of freedom and *P* value noted<br>*Give P values as exact values whenever suitable.*
☒	☐	For Bayesian analysis, information on the choice of priors and Markov chain Monte Carlo settings
☒	☐	For hierarchical and complex designs, identification of the appropriate level for tests and full reporting of outcomes
☒	☐	Estimates of effect sizes (e.g. Cohen's *d*, Pearson's *r*), indicating how they were calculated

Our web collection on statistics for biologists contains articles on many of the points above.

Software and code

Policy information about availability of computer code

Data collection	LabView2014 was used to synchronously control the laser power, photon counting, galvo mirror and motorized stage.
Data analysis	ImageJ/Fiji (2021) was used to process the imaging data obtained by our NIR imaging system.<br>Origin 2021 (OriginLab) was used to draw the curves and analyze the standard deviation.<br>Jade 6.5.26 was used to process and analyze the original XRD data.<br>FlowJo software v.10.8.1 was used to process and analyse the original flow-cytometry data.<br>Statistical analysis was performed using GraphPad Prism 8.0.

For manuscripts utilizing custom algorithms or software that are central to the research but not yet described in published literature, software must be made available to editors and reviewers. We strongly encourage code deposition in a community repository (e.g. GitHub). See the Nature Portfolio guidelines for submitting code & software for further information.

Data

Policy information about availability of data

All manuscripts must include a data availability statement. This statement should provide the following information, where applicable:

- Accession codes, unique identifiers, or web links for publicly available datasets
- A description of any restrictions on data availability
- For clinical datasets or third party data, please ensure that the statement adheres to our policy

The main data supporting the results in this study are available within the paper and its Supplementary Information. Source data for Figs. 3a–e and for Extended Data Fig. 2c are provided with this paper. The raw and analysed datasets generated during the study are too large to be publicly shared, yet they are available for research purposes from the corresponding author on reasonable request.

Human research participants

Policy information about studies involving human research participants and Sex and Gender in Research.

Reporting on sex and gender	The study did not involve human research participants.
Population characteristics	—
Recruitment	—
Ethics oversight	—

Note that full information on the approval of the study protocol must also be provided in the manuscript.

Field-specific reporting

Please select the one below that is the best fit for your research. If you are not sure, read the appropriate sections before making your selection.

☒ Life sciences ☐ Behavioural & social sciences ☐ Ecological, evolutionary & environmental sciences

For a reference copy of the document with all sections, see nature.com/documents/nr-reporting-summary-flat.pdf

Life sciences study design

All studies must disclose on these points even when the disclosure is negative.

Sample size	As other studies in this research area, at least 5 mice per group were used for assessing the therapeutic efficacy of the nanovaccine, and 3 mice per group were determined to be sufficient for the reproducibility of molecular imaging, and 6 mice per group for the reproducibility of flow cytometry.
Data exclusions	No data were excluded from the analyses.
Replication	All experiments were carried out at least 3 times, with similar results.
Randomization	Mice were randomly selected from the cages, and divided into groups.
Blinding	The investigators were blinded to group allocation during data collection and/or analysis.

Reporting for specific materials, systems and methods

We require information from authors about some types of materials, experimental systems and methods used in many studies. Here, indicate whether each material, system or method listed is relevant to your study. If you are not sure if a list item applies to your research, read the appropriate section before selecting a response.

Materials & experimental systems

n/a	Involved in the study
☐	☒ Antibodies
☐	☒ Eukaryotic cell lines
☒	☐ Palaeontology and archaeology
☐	☒ Animals and other organisms
☒	☐ Clinical data
☒	☐ Dual use research of concern

Methods

n/a	Involved in the study
☒	☐ ChIP-seq
☐	☒ Flow cytometry
☒	☐ MRI-based neuroimaging

Antibodies

Antibodies used	Anti-mouse-CD3-FITC (BioLegend; Cat No. 100204; Clone 17A2; Lot No. B361053; Dilution 5ug/mL)<br>Anti-mouse-CD8a-BV421 (BioLegend; Cat No. 100737; Clone 53-6.7; Lot No. B358296; Dilution 0.5ug/mL)<br>Anti-CD8 cys-diabody provided by Anna M. Wu and Felix Salazar.
Validation	Anti-mouse-CD3-FITC. Manufacturer's website link: https://www.biolegend.com/en-us/products/fitc-anti-mouse-cd3-antibody-45<br>There are publications using anti-CD8 cys-diabody: Journal of Nuclear Medicine 56.8 (2015): 1258-1264; Scientific reports 11.1 (2021): 1-12; Angewandte Chemie 132.46 (2020): 20733-20741.<br><br>anti-mouse-CD8a-BV421. Manufacturer's website link: https://www.biolegend.com/en-us/products/brilliant-violet-421-anti-mouse-cd8a-antibody-7138

Eukaryotic cell lines

Policy information about cell lines and Sex and Gender in Research

Cell line source(s)	The cell lines E.G-7 OVA and EL4 were purchased from ATCC.
Authentication	The cell lines were not authenticated.
Mycoplasma contamination	The cell lines tested negative for mycoplasma contamination
Commonly misidentified lines (See ICLAC register)	No commonly misidentified cell lines were used.

Animals and other research organisms

Policy information about studies involving animals; ARRIVE guidelines recommended for reporting animal research, and Sex and Gender in Research

Laboratory animals	Female C57BL/6 mice 6–7 weeks of age and 15–20 g of weight were purchased from Charles River.
Wild animals	The study did not involve wild animals.
Reporting on sex	The choice of sex was arbitrary.
Field-collected samples	The study did not involve samples collected from the field.
Ethics oversight	All procedures performed on the mice were approved by Stanford University's Institutional Animal Care and Use Committee.<br>All experiments were performed according to the National Institutes of Health Guide for the Care and Use of Laboratory Animals. The laboratory animal care program at Stanford is accredited by the Association for the Assessment and Accreditation of Laboratory Animal Care.

Note that full information on the approval of the study protocol must also be provided in the manuscript.

Flow Cytometry

Plots

Confirm that:

☒ The axis labels state the marker and fluorochrome used (e.g. CD4-FITC).

☒ The axis scales are clearly visible. Include numbers along axes only for bottom left plot of group (a 'group' is an analysis of identical markers).

☒ All plots are contour plots with outliers or pseudocolor plots.

☒ A numerical value for number of cells or percentage (with statistics) is provided.

Methodology

Sample preparation

1. Flow-cytometry analysis of antigen-specific CD8+ cells in tumour
Tumour tissue was harvested in cold PBS and digested by cutting into ~2 mm3 pieces and treated with DNAase I (StemCell Inc., 07900) and collagenase/hyaluronidase (StemCell Inc., 07912) at 37C for 25 mins with shaking. The dissociated tumour was passed through a 70-µm filter to obtain a single-cell suspension. Leukocyte-enriched fraction is then prepared using Mouse TIL CD45 positive selection kit (StemCell Inc., 100-0350). Cells were resuspended in RPMI media with 10% heat-inactivated FBS and 1% L-glutamine and recovered at 37 °C for 30 min with or without 50nM dasatinib (PKI). Cells were then labeled with PE-conjugated H-2Kb-SIINFEKL tetramer at 37 °C for 30mins. After tetramer labeling, cells were stained with anti-mouse-CD3-FITC, anti-mouse-CD8a-BV421 and Live/Dead NIR dye for flow-cytometry analysis.

2. Cell sorting of lymph-node immune cells
The inguinal lymph nodes (iLNs) were harvested and mechanically dissociated using a syringe rubber head in complete RPMI media supplemented with 10% HI FBS, 2 mM glutamine, and 1% penicillin/streptomycin containing 25 mM HEPES. The tissue suspension was passed through a 70-µm cell strainer to obtain a single-cell suspension. The cells were washed once by being centrifuged at 300 × g for 5 minutes at 4°C, and resuspended in cold PBS with 1% FBS for subsequent staining. For surface staining for FACS, the cells were incubated with an antibody/dye mix containing anti-mouse CD3-AF488 (pan T cells), anti-mouse CD19-PE (B cells), anti-mouse CD11c-PE/Cy7 (dendritic cells), anti-mouse F4/80-BV421 (macrophages), Live/Dead Fixable Near IR dye, and TruStain FcX PLUS (anti-mouse CD16/32 for Fc receptor blockade) (BioLegend) for 15 minutes at 4°C in dark. After incubation, cells were washed with cold PBS containing 1% FBS, then resuspended in cold PBS containing 1% FBS and 2 mM EDTA for sorting. Cells were filtered with 70-µm strainers right before sorting to remove clumps and cell aggregates.

3. Flow-cytometry analysis of the EG-7 cell line
To perform flow-cytometry analysis, th eEG-7 cell suspension was centrifuged at 400 × g for 5 minutes at 4°C. The supernatant was then discarded, and the cell pellet was resuspended in staining buffer (PBS with 0.5% BSA) at a concentration of 1×10^6 cells/ml. The cell suspension was divided into aliquots for different staining panels or controls. Fluorochrome-conjugated antibodies specific for CD3 (PE-conjugated, 1:50 dilution; BioLegend), CD4 (APC-conjugated, 1:50 dilution; BioLegend), and anti-mouse CD8a (FITC-conjugated, 1:50 dilution; Invitrogen) antigens were added to the cell suspension. The cells were incubated with the antibodies for 30 minutes at 4°C, protected from light. After incubation, the cells were washed with staining buffer and centrifuged at 400 × g for 5 minutes at 4°C. Flow-cytometry data were acquired using a Beckman CytoFLEX flow cytometer and analyzed using FlowJo software (version 10.8.1).

Instrument

BD LSRFortessa flow cytometer; BD FACS Aria II sorter; Beckman CytoFLEX flow cytometer.

Software

FlowJo software v.10.8.1.

Cell population abundance

Cell sorting of lymph-node immune cells was performed at 4C using a BD FACS Aria II sorter.

Gating strategy

Based on the pattern of FSC-A/SSC-A, cells in the lymphocyte gate were identified. Singlets were then identified by the pattern of FSC-A/FSC-H and SSC-A/SSC-H. As followed, viable T cells were determined as the Live/Dead NIR negative and CD3 positive populations. Cells in the viable T-cell gate were analysed for CD8a expression and tetramer binding. Positive gate boundaries were determined by the distinction from the fluorescence Minus One (FMO) controls of each channel.

☒ Tick this box to confirm that a figure exemplifying the gating strategy is provided in the Supplementary Information.
